# Cross-infectivity of honey and bumble bee-associated parasites across three bee families

**DOI:** 10.1017/S0031182020001018

**Published:** 2020-10

**Authors:** Lyna Ngor, Evan C. Palmer-Young, Rodrigo Burciaga Nevarez, Kaleigh A. Russell, Laura Leger, Sara June Giacomini, Mario S. Pinilla-Gallego, Rebecca E. Irwin, Quinn S. McFrederick

**Affiliations:** 1 Department of Entomology, University of California Riverside, Riverside, CA, USA; 2 Department of Applied Ecology, North Carolina State University, Raleigh, NC 27695, USA

**Keywords:** Alfalfa leafcutter bee, blue orchard bee, flagellate, *Halictus ligatus*, host–parasite specificity, Kinetoplastidae, Leishmaniiniae, *Megachile rotundata*, *Osmia lignaria*, sweat bee

## Abstract

Recent declines of wild pollinators and infections in honey, bumble and other bee species have raised concerns about pathogen spillover from managed honey and bumble bees to other pollinators. Parasites of honey and bumble bees include trypanosomatids and microsporidia that often exhibit low host specificity, suggesting potential for spillover to co-occurring bees *via* shared floral resources. However, experimental tests of trypanosomatid and microsporidial cross-infectivity outside of managed honey and bumble bees are scarce. To characterize potential cross-infectivity of honey and bumble bee-associated parasites, we inoculated three trypanosomatids and one microsporidian into five potential hosts – including four managed species – from the apid, halictid and megachilid bee families. We found evidence of cross-infection by the trypanosomatids *Crithidia bombi* and *C. mellificae*, with evidence for replication in 3/5 and 3/4 host species, respectively. These include the first reports of experimental *C. bombi* infection in *Megachile rotundata* and *Osmia lignaria*, and *C. mellificae* infection in *O. lignaria* and *Halictus ligatus*. Although inability to control amounts inoculated in *O. lignaria* and *H. ligatus* hindered estimates of parasite replication, our findings suggest a broad host range in these trypanosomatids, and underscore the need to quantify disease-mediated threats of managed social bees to sympatric pollinators.

## Introduction

Host mobility and interspecific host contact create the potential for transmission of parasites among populations of different hosts. Few systems exemplify the principles of host mobility and resource sharing to the extent of plant–pollinator interaction networks. Pollinating bees are highly mobile, capable of visiting thousands of flowers per day at a variety of distances from their nest sites (Heinrich, 2004; Greenleaf *et al*., 2007). Many species are also generalists that collect nectar and pollen from a wide variety of floral species, resulting in interspecific niche overlap and visits of different types of bees to the same flowers over short periods of time (Heinrich, 1976*a*; Goulson and Darvill, 2004; Ruiz-González *et al*., 2012). Bees host a diverse assemblage of parasites (Evans and Schwarz, 2011), many of which are transmissible by fecal–oral routes (Durrer and Schmid-Hempel, 1994; Graystock *et al*., 2015; Engel *et al*., 2016). In particular, the large colonies of social honey bees (*Apis* spp.) and bumble bees (*Bombus* spp.) have high potential to spread parasites to other individuals and species by deposition at flowers, which can serve as sites of transmission (Durrer and Schmid-Hempel, 1994; McArt *et al*., 2014; Graystock *et al*., 2015; Adler *et al*., 2018). These circumstances create potential for parasite transfer within plant–pollinator networks, and favour parasites that can exploit multiple hosts. Moreover, aside from local transmission, the global trade and seasonal movement of agriculturally managed bees creates unprecedented opportunities for parasites to invade new host populations (Graystock *et al*., 2016). Examples of human-assisted parasite invasions include the spread of the *Varroa* mite, its associated viruses and the microsporidian *Nosema ceranae* (Fries *et al*., 1996) in honey bees (Klee *et al*., 2007; Rosenkranz *et al*., 2010) and the spread of the trypanosomatid *Crithidia bombi* (Lipa and Triggiani, 1988) to South American bumble bees (Arbetman *et al*., 2012; Schmid-Hempel *et al*., 2014).

Managed pollinators such as European honey bees (*Apis mellifera* Linnaeus) and, more recently, bumble bees (*Bombus impatiens* Cresson and *Bombus terrestris* Linnaeus) (Velthuis and van Doorn, 2006) have been shown to harbour bacterial, fungal, protozoal and viral infections (Schmid-Hempel, 1998; Cornman *et al*., 2012; Vanbergen and Insect Pollinators Initiative, 2013). Wild bee populations may also host viral (Dolezal *et al*., 2016), bacterial (Li *et al*., 2015), microsporidian (Fantham and Porter, 1914), trypanosomatid (Schmid-Hempel, 2001) and nematode (McFrederick *et al*., 2013) infections. However, relatively little is known about the parasites of most bee species (Goulson, 2003; Goulson *et al*., 2015). Parasites associated with managed honey bees and bumble bees have been detected at flowers and in association with alternative hosts (i.e. hosts other than the ‘primary’ genus or species with which the parasite is traditionally associated) (Ravoet *et al*., 2014; McMahon *et al*., 2015; Alger *et al*., 2019). In a few cases, parasites have remained detectable for days following experimental inoculation, and occasionally elevated mortality of alternative hosts (Ruiz-González and Brown, 2006; Graystock *et al*., 2013; Fürst *et al*., 2014; Dolezal *et al*., 2016; Müller *et al*., 2019; Purkiss and Lach, 2019; Strobl *et al*., 2019). These studies suggest the potential for spillover of parasitic infection from honey bees and bumble bees to other managed and wild bees (Mallinger *et al*., 2017). In addition to uni-directional spillover, high densities of managed bees could contribute to amplification of infections with native or introduced multi-host parasites, which then ‘spill back’ into the original host populations (Graystock *et al*., 2016). However, despite its importance for disease epidemiology in bee communities, in most cases the extent of replication by bee parasites in alternative hosts remains unknown. We aimed to build on field-based molecular surveys of host–-parasite associations by measuring parasite persistence and replication under controlled conditions in experimentally inoculated bees.

**Fig. 1.**
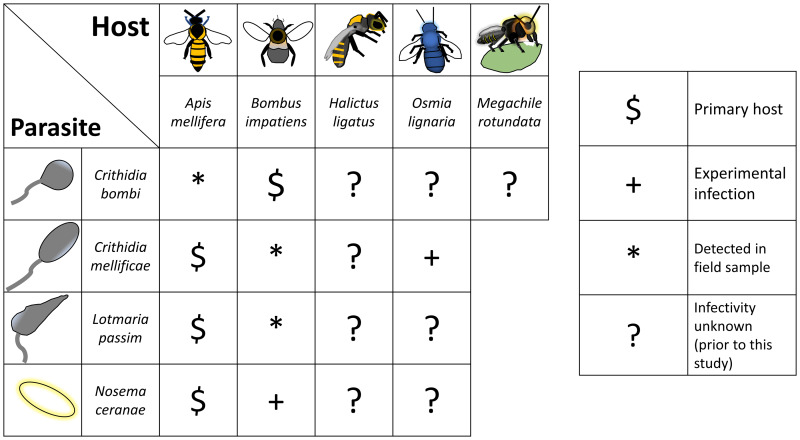
Schematic of experimental design, indicating host–parasite combinations tested and previously documented infectivity. Dollar sign (‘$’) indicates recognized (‘primary’) host. Plus sign (‘+’) indicates experimental infection of a congeneric host species in at least one study. Asterisk (‘*’) indicates detection in field samples. Question mark (‘?’) indicates that infectivity was unknown prior to this study. See Materials and methods: Study system for references that document infection.

To better understand the infectivity of honey and bumble bee-associated parasites in alternative hosts, we inoculated four parasite species (three trypanosomatids and one microsporidian) into five bee species (two managed and introduced, two native and managed, one native and unmanaged in North America) from three different bee families (Apidae, Halictidae and Megachilidae) (Fig. 1). Each experiment included a primary host species as a positive control for parasite infectivity and – for all but one host–parasite combination – also included sham-inoculated negative controls to screen for pre-existing infection and effects of parasites on short-term mortality. We defined ‘infection’ as within-host parasite replication, based on an unambiguous increase in the number of parasites post-inoculation. We defined ‘persistence’ of parasites for cases where parasites remained detectable in the gut for longer than expected based on observed gut transit times. This persistence could facilitate the spread – or ‘vectoring’ – of parasites by transient hosts in which parasites survive, but replication does not occur (Ruiz-González and Brown, 2006). We predicted that more closely related hosts (i.e. *B. impatiens* and *A. mellifera*) would be more likely to share parasites. We also expected that extracellular parasites (trypanosomatids) would show lower host specificity than would intracellular parasites (microsporidia), reflecting the complex and host-specific needs of intracellular species [e.g. to enter and exit host cells and attenuate intracellular immune defenses (Sibley, 2011)]. Our study helps to define the host range and spillover potential of parasites associated with honey and bumble bees into alternative hosts.

## Materials and methods

### Study system

We chose four common bee parasites (Fig. 1) based on ease of inoculation and ability to spread *via* shared flowers (Durrer and Schmid-Hempel, 1994; Graystock *et al*., 2015; Adler *et al*., 2018), and potential for cross-species transmission. Insect-associated trypanosomatids in general have low host specificity (Wallace, 1966; Kozminsky *et al*., 2015); honey bee-associated *Nosema* spp. spores are both infective and virulent in insects from several different taxonomic orders, including Hymenoptera, Lepidoptera and Diptera (Fantham and Porter, 1913).

The trypanosomatid *C. bombi* is traditionally associated with *Bombus* spp. in Europe (Lipa and Triggiani, 1988). However, the parasite is geographically widespread, having been documented in *Bombus* spp. of Europe, North America and South America (Schmid-Hempel and Tognazzo, 2010; Schmid-Hempel *et al*., 2014). A single strain can infect multiple European *Bombus* spp. (Ruiz-González *et al*., 2012; Schmid-Hempel *et al*., 2014), and local prevalence can exceed 50% among worker bees in both Europe and North America (Schmid-Hempel, 2001; Gillespie, 2010), suggesting this parasite's potential to infect diverse hosts. The parasite has a range of effects on hosts in its presumed native and introduced ranges. These include elevated queen mortality (Brown *et al*., 2003; Fauser *et al*., 2017), reduced tolerance to food deprivation (Brown *et al*., 2000) and reduced colony size and reproduction in European *B. terrestris* (Brown *et al*., 2003), and reduced foraging rate in North American *B. impatiens* (Otterstatter *et al*., 2005). Field samples have detected *C. bombi* in the *Bombus* subgenus *Psithyrus* spp. in Switzerland (Schmid-Hempel and Tognazzo, 2010) and *A. mellifera* in Spain (Bartolomé *et al*., 2018). However, experimental inoculations of *A. mellifera* with *C. bombi* indicated that although parasites remained viable in the gut and feces for up to 7 day post-inoculation, quantities were below the levels used for inoculation, indicating a lack of successful infection (Ruiz-González and Brown, 2006).

We also conducted experimental inoculations with two other trypanosomatids, *Crithidia mellificae* (Langridge and McGhee, 1967) and *Lotmaria passim* (Schwarz *et al*., 2015). *Crithidia mellificae* is a confirmed multi-host parasite, with the type strains isolated from *A. mellifera* and the yellow jacket *Vespula squamosa* (Langridge and McGhee, 1967; Schwarz *et al*., 2015). Experimental inoculations have indicated infectivity in *Osmia cornuta* and *Osmia bicornis* (Schwarz *et al*., 2015; Strobl *et al*., 2019). Molecular analyses of field samples have also detected the parasite in *B. terrestris* (Bartolomé *et al*., 2018). Previously not distinguished from *C. mellificae*, *L. passim* is the recently described parasite species now believed to be the dominant trypanosomatid in honey bees worldwide (Ravoet *et al*., 2015; Schwarz *et al*., 2015) and correlated with collapse of colonies (Cornman *et al*., 2012). Thus far, infectivity has only been shown experimentally in *A. mellifera* (Schwarz *et al*., 2015; Liu *et al*., 2020), but the parasite was detectable by polymerase chain reaction (PCR) in field samples of *B. terrestris* (Bartolomé *et al*., 2018).

The microsporidian *N. ceranae* (Fries *et al*., 1996), believed to originate from the Asian honey bee (*Apis cerana*), has replaced *Nosema apis* as the dominant honey bee microsporidian worldwide (Klee *et al*., 2007; Paxton *et al*., 2007). Infection has been linked to colony death (Higes *et al*., 2008; Cornman *et al*., 2012) and a variety of sublethal effects (Fries *et al*., 2013), including midgut lesions (Higes *et al*., 2008), immunosuppression (Antúnez *et al*., 2009) and reduced hypopharyngeal gland development (Jack *et al*., 2016). Experimental inoculations have indicated infectivity and virulence in *B. terrestris* (Graystock *et al*., 2015) – although other studies showed no evidence of infection (Piiroinen *et al*., 2016; Gisder *et al*., 2020) – and in the stingless bee *Tetragonula hockingsi* (Purkiss and Lach, 2019). *Nosema ceranae* was also detected by PCR in several wild South American *Bombus* spp. (Plischuk *et al*., 2009).

We tested the infectivity of these parasites in five potential hosts of the Apidae, Megachilidae and Halictidae families. Host bee species were chosen based on taxonomic breadth, use and distribution for agriculture and availability; all are widespread generalist foragers that are likely exposed to honey and bumble bee-associated parasites at shared flowers in wild and agricultural settings. In addition to managed *B. impatiens* and *A. mellifera* (both in the family Apidae), we used the semi-managed Megachilid species *Megachile rotundata* Fabricius (alfalfa leafcutter bee) and *Osmia lignaria* Say (blue orchard bee). Both megachilids are solitary, cavity-nesting species. Their dormant overwintering life stages are collected in trap nests that are widely distributed for pollination of orchard and forage crops (Pitts-Singer and Cane, 2011; Boyle and Pitts-Singer, 2019). To our knowledge, neither species has been tested for susceptibility to trypanosomatids or microsporidia. Finally, we included the Halictid *Halictus ligatus* Say (ligated furrow bee), an example of an unmanaged but widely distributed, generalist forager, in which microparasites have likewise received little attention. Of these species, *A. mellifera* and *M. rotundata* are native to Europe, whereas *B. impatiens*, *H. ligatus* and *O. lignaria* are native to North America. For brevity, bee species are referred to by their genus names (*Apis*, *Bombus*, *Halictus*, *Megachile* and *Osmia*) in figures.

### Experimental design

We conducted two sets of experiments, each of which involved oral inoculation of bees with purified parasites, rearing for 7–8 days under controlled conditions to allow development of infection, and subsequent dissection and parasite quantification. All bees except for the wild-collected *H. ligatus* emerged and were reared in the laboratory to reduce the chance of pre-existing infection. The first set of experiments (summer 2018), hereafter referred to as the ‘*C. bombi–Megachile* experiment’, tested infectivity of *C. bombi* in *M. rotundata* using microscopic quantification of infection intensity. This method allowed us to assess parasite replication in both a primary *Bombus* host (*B. impatiens*) and an alternative *Megachile* host (*M. rotundata*). A second, larger series of experiments – hereafter referred to as the ‘factorial cross-infection experiment’ – was conducted with additional parasites and hosts. The factorial cross-infection experiment used molecular quantification of parasites by quantitative PCR (qPCR), and included sham-infection (i.e. negative) control treatments to assess effects of parasite inoculation on host mortality and pre-existing infection not due to our inoculations. This experiment was run in four blocks, each of which tested the effects of a single parasite species on four host species. Our original intention was to use a fully crossed design in which each bee species was inoculated with each parasite species. However, due to limited emergence and survival of *O. lignaria* for experiments with *C. bombi* and *L. passim*, inoculations with *C. bombi* and *L. passim* were repeated the following summer (2019) with *O. lignaria* only.

### Sources of bees

*Apis mellifera* workers were obtained from a colony at the University of California Riverside. Brood frames were collected 4 days prior to inoculation and placed in an incubator (30°C). Newly emerged workers were collected the following morning (i.e. 3 days pre-inoculation) and reared together in wire mesh cages in groups of ~30 bees with *ad libitum* access to 50% sugar water and pollen (Brushy Mountain Bee Farms, Moravia Falls, NC; used for all rearing and experiments).

*Bombus impatiens* colonies were obtained from Koppert Biological Supply (Howell, MI). Colonies were reared at 27°C (or 21–25°C for colonies used in the *C. bombi*–*Megachile* experiment) with *ad libitum* access to 50% sugar water (or 30% sugar for colonies in *C. bombi*–*Megachile* experiment) and pollen. Worker bees from one colony were used for the *C. bombi*–*Megachile* experiment; seven additional colonies (five per experiment) were used for the factorial cross-infection experiment. We acknowledge that susceptibility to trypanosomatid and microsporidian infection can vary across colonies (Koch and Schmid-Hempel, 2012; Chaimanee *et al*., 2013; Barribeau *et al*., 2014), and that our experiments cannot rule out the possibility of greater or lesser infection in conspecific bees of other colonies.

*Halictus ligatus* were collected from a wild aggregation at the Hidden Valley Nature Center (Jurupa Valley, CA, GPS coordinates: 33.96, −117.50) 3 days prior to inoculation and housed in groups of 30–40 bees in mesh rearing cages with access to 50% sugar water and pollen. The species is not endangered, and no permits were required for collection.

*Osmia lignaria* and *M. rotundata* were obtained from Watt's bees (Bothell, WA) in the overwintered pharate state (i.e. adult bees still inside their pupal cocoons) for *O. lignaria* and the prepupal stage for *M. rotundata*. Cocoons were stored at 4°C until 5 days (*O. lignaria*) or 6 weeks (*M. rotundata*) before the experiments. To stimulate emergence of *O. lignaria* adults, cocoons were moved to a 32°C incubator. Upon emergence, bees were moved to individual 60 mL plastic cups and given *ad libitum* access to 50% sugar water and pollen until 24 h pre-inoculation. To stimulate emergence of *M. rotundata*, cocoons were incubated at room temperature (21–25°C) until adults emerged, then transferred to a 60 × 60 × 60 cm^3^ cage, where they were held under ambient lab conditions for ~2 days prior to inoculations.

For *H. ligatus* and *O. lignaria*, a mixture of male and female bees was used. Sex ratios were generally imbalanced and reflective of ratios in the random sampling of field-collected bees (*H. ligatus)* or the recently emerged laboratory-reared cohort (*O. lignaria*). Males and females were differentiated by post-experiment microscopy of sexually dimorphic characteristics (antennal colour pattern in *H. ligatus*, antennal length and copious pale facial hairs on males *O. lignaria*). Sex-specific sample sizes for *H. ligatus* and *O. lignaria* are summarized in Supplementary Table 1.

### Parasites

For the *C. bombi–Megachile* experiment, gut homogenates of infected bees were used. The infection originated from wild *B. impatiens* workers collected near Amherst, Massachusetts, USA (GPS coordinates: 42°22′17.53″N, 72°35′13.52″W). The infection was established in commercial colonies by feeding gut homogenate of the infected wild bees to workers of the commercial colony, then serially transferred to younger colonies every 4–6 weeks by the same procedure. Species identity was confirmed by sequencing of the 18s rRNA gene (Schmid-Hempel and Tognazzo, 2010; Figueroa *et al*., 2019).

For the factorial cross-infection experiment, we used axenic trypanosomatid cell cultures rather than gut homogenates. Strains of bee-infective trypanosomatids – most notably *C. bombi* – can vary in infectivity (Sadd and Barribeau, 2013; Barribeau *et al*., 2014). To reduce the chances of false negatives due to strain-specific incompatibility with alternative hosts, and more closely approximate the mixture of strains to which bees would likely be exposed in naturally diverse parasite populations (Salathé *et al*., 2012), we inoculated bees with mixtures of several parasite strains. Three strains of *C. bombi* were isolated from infected wild bumble bees (*B. impatiens* and *B. terrestris*) by single cell sorting: strains ‘12.6’ (from *B. impatiens* in Lufkin, TX in 2014 by Hauke Koch), ‘IL13.2’ (from *B. impatiens* in Normal, IL in 2013 by Ben Sadd) and ‘C1.1’ (from *B. terrestris* in Corsica, France in 2012 by Ben Sadd) (Palmer-Young *et al*., 2016). Species identity was confirmed by sequencing of the GAPDH gene (Palmer-Young *et al*., 2019*a*, 2019*b*). Parasites were grown at 27°C in vented culture flasks with modified Mattei growth medium as previously described (Salathé *et al*., 2012). *Crithidia mellificae* [ATCC cultures 30254 from *A. mellifera* and 30862 from *V. squamosa* (Langridge and McGhee, 1967)] and *L. passim* [ATCC cultures PRA-403 (strain ‘SF’) and PRA-422 (strain ‘BRL’) (Schwarz *et al*., 2015)] were obtained from the American Type Culture Collection. Cell cultures of all three trypanosomatids (*C. bombi*, *C. mellificae* and *L. passim*) were cryopreserved at −80°C until 3–5 days prior to inoculations, then grown at 27°C in vented 25 cm^2^ culture flasks with a modified Mattei medium containing 10% heat-inactivated fetal bovine serum (Salathé *et al*., 2012).

*Nosema ceranae* was obtained from infected *A. mellifera* from the University of California San Diego. The parasites originated from *A. cerana* and *A. florea* from Thailand; species identity was confirmed as *N. ceranae* by sequencing the PCR product obtained from species-specific primers for the RPB1 gene (Eiri *et al*., 2015). The parasites were passaged every 10 days by feeding purified spore suspensions [purified from the guts of infected *A. mellifera* by Percoll density gradient (Fries *et al*., 2013), see ‘Inoculation’ section] to *A. mellifera* workers from colonies at the University of California Riverside.

### Inoculation

#### Trypanosomatids

**Fig. 2.**
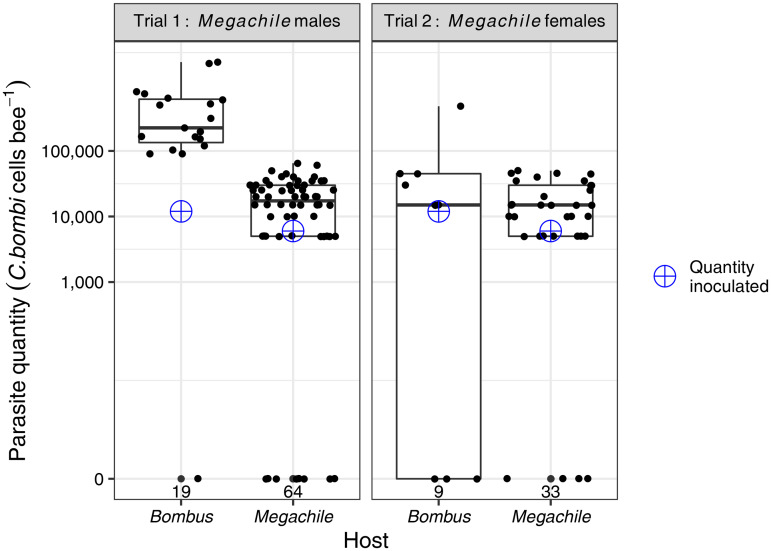
Infectivity of *C. bombi* in *M. rotundata* and the original host, *B. impatiens*. Boxplots show median (dark middle line) and interquartile range (upper and lower bounds of box). Whiskers extend to the most extreme data point within 1.5 times the interquartile range of the first or third quartile. Points show estimated parasite quantities of each individual based on microscopic cell counts, randomly offset to the left and right to avoid overplotting. Hatched circles indicate the number of cells with which bees were inoculated (12 000 for *B. impatiens*, 6000 for *M. rotundata*). Note the log scale on the *y*-axis. Numbers along the *x*-axis indicate sample sizes.

In the *C. bombi–Megachile* experiment, a parasite-containing inoculum was prepared from gut homogenates of bees from infected colonies. In the first trial, male *M. rotundata* (*n* = 64, Fig. 2) were inoculated with 6000 parasite cells in 5 *μ*L 25% sugar water. Concentrations in the inoculum were designed to mimic those in a ~10-fold dilution of infected *B. impatiens* feces (Otterstatter and Thomson, 2006), as might be encountered by bees foraging at floral nectaries, and are fairly standard for experiments with *C. bombi* and *L. passim* (Barribeau *et al*., 2014; Schwarz *et al*., 2016). Half the bees received an inoculum prepared from infected bumble bee feces, diluted in distilled water. The other half received an inoculum from infected bumble bee gut homogenates. Gut homogenates were centrifuged three times (15′, 2000 rpm) to pellet the parasites. After each centrifugation, the supernatant was removed and the pellet resuspended in deionized water. In the second trial, female *M. rotundata* (*n* = 33) were inoculated with inoculum prepared from diluted homogenized, settled (4 h) gut extracts, without centrifugation (Richardson *et al*., 2015). On each inoculation date, 10 *B. impatiens* from commercial colonies were inoculated with 10 *μ*L (12 000 cells) of the same inoculum used for *M. rotundata* inoculation; this larger quantity was used due to the larger size of *B. impatiens* relative to *M. rotundata*. These *B. impatiens* served as positive controls to confirm the infectivity of the inoculum. The *C. bombi–Megachile* experiments did not include negative controls (i.e. bees inoculated with a sham inoculum that contained no parasites).

For trypanosomatid inoculations with *C. bombi*, *C. mellificae* and *L. passim* in the factorial cross-infection experiment, cell cultures were diluted to 2000 cells *μ*L^−1^ in growth medium. The inoculum was composed of equal concentrations of the three (*C. bombi*) or two (*C. mellificae* and *L. passim*) parasite strains (e.g. partial concentrations of 1000 cells *μ*L^−1^ each of two parasite strains). The cell suspension was then mixed with an equal quantity of 4 mm (*C. mellificae*) or 16 mm (*L. passim* and *C. bombi*) aqueous sucralose (trade name ‘Splenda’, Heartland Food Products, UK) water for a final concentration of 1000 parasites *μ*L^−1^. The sucralose solution was used to provide a sweet taste that encouraged consumption without the osmotic stress of a high-sugar solution (Palmer-Young *et al*., 2019*a*, 2019*b*), which can kill trypanosomatid cells (Cisarovsky and Schmid-Hempel, 2014); we observed that cells rapidly became deformed and immotile in 50% sugar water. The higher 16 mm sucralose concentration (8 mm in final inoculum) was used in the final 2 weeks of the experiment after this concentration was found to promote consumption. For all treatments, the sucralose solution was coloured with 0.1% red #40 food dye, which made it easier to track whether bees had been successfully inoculated. *Bombus impatiens* were fed with a 10 *μ*L droplet of inoculum (10 000 cells) from a micropipette. *Halictus ligatus* and *O. lignaria* would not consume parasite-containing solutions on demand, so we were unable to hand-inoculate them, which prevented quantification of the number of parasites consumed by each bee. Instead, bees of these species were isolated in individual 60 mL plastic cups (one per bee) and allowed to feed overnight from tubes containing ~200 *μ*L of the coloured inoculum. A large hole was made in each tube using a soldering iron to improve the likelihood that bees would encounter, recognize and consume the inoculum. Attempts to estimate quantities inoculated by measuring consumption were unsuccessful due to the relatively large surface area of the soldered drinking hole, which led to substantial and variable losses due to handling, evaporation and occasional splashing of the tube's contents by experimental bees, which was reflected by the pattern of red stains that appeared in the rearing cup.

**Fig. 3.**
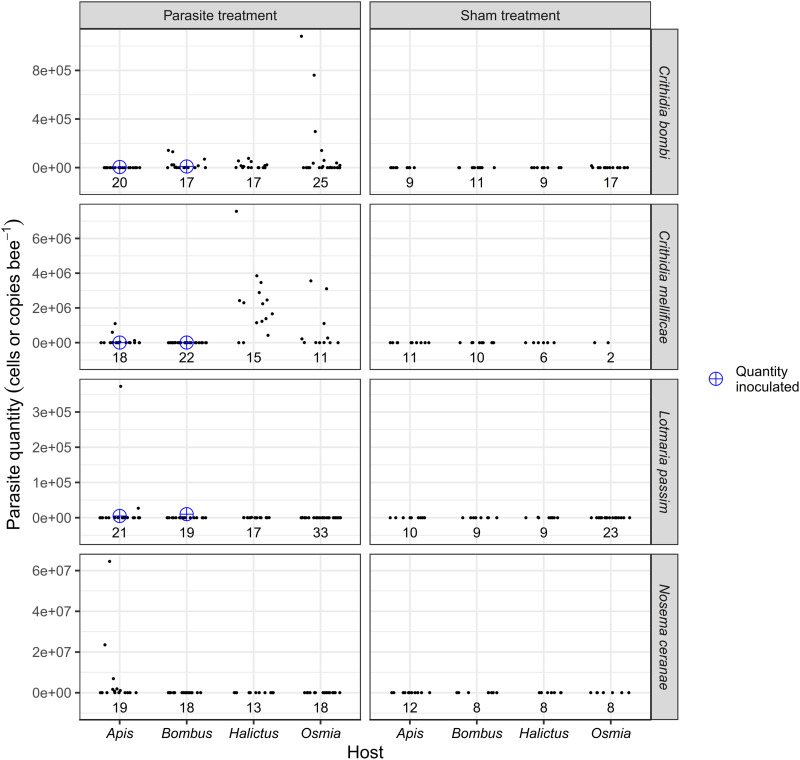
Infectivity of four parasites across bee species of three families: *A. mellifera* (Apidae), *B. impatiens* (Apidae), *H. ligatus* (Halictidae) and *O. lignaria* (Megachilidae). Points show estimated parasite quantities of each individual based on qPCR, randomly offset to the left and right to avoid overplotting. The *y*-axis for each parasite corresponds to standards used in qPCR (cell equivalents for the trypanosomatids *C. bombi*, *C. mellificae* and *L. passim*; plasmid copy equivalents for the microsporidian *N. ceranae*). Samples with Cq > 40 are plotted as zeroes. Hatched circles indicate the number of cells with which bees were inoculated (10 000 for *B. impatiens*, 5000 for *A. mellifera*, not quantified for *H. ligatus* or *O. lignaria*). Numbers along the *x*-axis indicate sample sizes.

*Apis mellifera* refused to consume solutions sweetened only with sucralose. They were instead fed a 10 *μ*L droplet containing 5000 parasite cells, consisting of 1 part parasite cell suspension:1 part sucralose solution:1 part 50% sugar water. We inoculated *A. mellifera* with half the number of cells used for *B. impatiens* to account for the relatively small size of *A. mellifera*. Bees in the sham infection treatment were treated and fed identically, but with parasite-free sham inoculum. Sample sizes are shown in Fig. 3 and Supplementary Table 2 for parasite quantification and in Supplementary Fig. 3 for survival.

#### Nosema ceranae

Spores from gut homogenates of infected *A. mellifera* were purified by Percoll gradient-based centrifugation (Fries *et al*., 2013). The spore suspension was diluted to 2000 cells *μ*L^−1^ in 0.01 m NH_4_Cl, then mixed with an equal volume of 50% sugar water to yield a final concentration of 1000 spores *μ*L^−1^. *Apis mellifera* and *B. impatiens* were inoculated from a micropipette with 5 *μ*L (5000 cells, *A. mellifera*) or 10 *μ*L (10 000 spores, *B. impatiens*) of the inoculum. These amounts represent ~10% of the amount found in a fecal dropping from infected *A. mellifera* (Copley *et al*., 2012) and exceed the estimated 85 spores per bee necessary to infect 50% of *A. mellifera* – 1000 spores was sufficient to infect 80% of bees, and 10 000 spores resulted in 100% infection (Forsgren and Fries, 2010). *Halictus ligatus* and *O. lignaria* did not consume solutions on demand, and were instead allowed to feed for 48 h (*H. ligatus*) or 24 h (*O. lignaria*) from microcentrifuge tubes containing 200 *μ*L of the 1000 cells *μ*L^−1^ spore suspension/sugar water solution. Bees in the sham infection treatment were treated and fed identically, but with parasite-free sham inoculum. Sample sizes are shown in Fig. 3 and Supplementary Table 2 for infection, and in Supplementary Fig. 3 for survival.

### Experimental bee rearing conditions

*Megachile rotundata* inoculated with *C. bombi* (and corresponding *B. impatiens* controls) were reared in individual 18.5 mL snap-cap vials, and fed pollen paste and 30% sucrose *ad libitum* from a 1.7 mL feeder tube with a cotton wick, inserted into the vial lid (Biller *et al*., 2015). Bees were incubated in a dark room at ambient temperature (21–25°C), checked daily for survival and dissected at 7 days post-inoculation, by which time trypanosomatid infections in the primary host *B. impatiens* are well developed (Otterstatter and Thomson, 2006).

In the factorial cross-infection experiment, *A. mellifera* were kept in groups of 30 bees in wire mesh cages and incubated at 35°C. All bees of a given block and infection treatment were housed in the same cage; we acknowledge that this results in pseudoreplication of bees within each cage. Sugar water (50% sucrose water) and pollen paste were provided *ad libitum*. *Bombus impatiens*, *H. ligatus* and *O. lignaria* were reared in individual 60 mL plastic cups at 30°C. All bees were given *ad libitum* access to a 1.7 mL feeder tube containing 50% sucrose water and a ~100 mg lump of pollen paste. Sugar water tubes were replaced daily or as needed. All groups were checked daily for survival. At 8 days post-inoculation, or on the date they were first observed dead, bees were frozen on dry ice in microcentrifuge tubes, then stored at −80°C until dissection. Trypanosomatid infections in *B. impatiens* are generally fully developed by this time (Otterstatter and Thomson, 2006), and *N. ceranae* quantities in *A. mellifera* have increased by 20- to 100-fold (Paxton *et al*., 2007; Martín-Hernández *et al*., 2009; Forsgren and Fries, 2010). Although *N. ceranae* spore production may continue to increase beyond this time (Forsgren and Fries, 2010), alternative hosts can exhibit 50–90% mortality within 5 days of inoculation (Graystock *et al*., 2013; Purkiss and Lach, 2019). Even *A. mellifera* can suffer 90–100% mortality within 10–14 days of *N. ceranae* inoculation (Higes *et al*., 2007; Dussaubat *et al*., 2013), with >50% mortality possible even without parasite inoculation (Eiri *et al*., 2015). Therefore, we terminated the experiment after 8 days to allow replication (or clearance) of parasites, but avoid excessive host mortality and consequent reduction in sample sizes.

### Dissection

Guts of *C. bombi-*inoculated *M. rotundata* (and *B. impatiens* controls) were homogenized using a disposable plastic pestle in 100 *μ*L (*M. rotundata*) or 300 *μ*L (*B. impatiens*) deionized water in a microcentrifuge tube. The homogenized bee guts were allowed to settle for 4 h, at which time infection intensity was quantified microscopically by counting cells from a 10 *μ*L aliquot of the resulting supernatant on a Neubauer hemocytometer. This procedure likely underestimates total parasite quantities by selecting for motile forms of the parasite over non-motile ‘spheroid’ or ‘amastigote’ forms (Logan *et al*., 2005; Schwarz *et al*., 2015). However, concentrations of *C. bombi* in supernatants of gut homogenate correlate well with concentrations of parasites in feces, thereby providing a conservative estimate of parasite quantities and a good proxy for infectiousness (i.e. the ability to spread parasites) (Otterstatter and Thomson, 2006). Parasites were visually identified by their characteristic shape and motility. All cells in a 0.02 *μ*L volume were counted under 400×  magnification (Richardson *et al*., 2015). The total number of parasite cells in each bee gut was estimated by multiplying the concentration of parasites in the supernatant by the total volume of gut homogenate. The intertegular distance (i.e. the distance between attachment points of the left and right forewings) was measured as an indicator of *M. rotundata* size.

Bees in the factorial cross-infection experiment were dissected to remove the mid- and hindgut using standard methods described in the BeeBook (Engel *et al*., 2013). Each individual body was surface-sterilized by rinsing for 3 min in 1% household bleach (0.05% sodium hypochlorite) and 3× 1 min in doubly deionized water. The gut was removed by pulling on the distal segment of the abdomen with sterile forceps and placed in a sterile tube for DNA extraction.

### DNA extraction and quality control

DNA of bees in the factorial cross-infection experiment was extracted using the Qiagen DNEasy blood and tissue kit (Qiagen, Hilden, Germany). Samples were treated with 180 *μ*L lysis buffer (Qiagen buffer ‘ATL’) and 20 *μ*L proteinase K solution, then homogenized for 6 min at 30 Hz in a TissueLyser (Qiagen) with a 3.2 mm diameter steel ball and 50 *μ*L of 0.1 mm glass beads. Homogenized samples were incubated overnight at 56°C in a convection oven. Subsequent DNA extraction was performed according to the manufacturer's instructions. Extracted DNA was stored at −80°C until use in PCR-based assays.

PCR of the Apidae 18S rDNA gene was used to confirm presence of host DNA. Assays were run with 10 *μ*L reaction volume, including 1 *μ*L template DNA, 200 nm each of forward and reverse primers [‘ApidaeF’ (AGATGGGGGCATTCGTATTG) and ‘ApidaeR’ (ATCTGATCGCCTTCGAACCT) (Meeus *et al*., 2010)], 200 nm of each dNTP, 1.5 mm MgCl_2_ [from 10× PCR buffer (New England Biolabs, Ipswich, MA)] and 0.25 units Taq DNA polymerase (New England Biolabs). Thermocycler conditions included 3 min denaturation (95°C), 34 cycles of 30 s at 95°C, 30 s at 57°C and 60 s at 72°C; and 5 min at 72°C. Products were visualized by gel electrophoresis on a 1.5% agarose gel and compared with those of a positive control sample that had amplified successfully in prior experiments. Samples that failed to amplify due to low purity or concentration were treated to remove excess guanidine or concentrate the DNA, respectively (see Supplementary Methods: DNA cleanup and concentration).

### Molecular quantification of parasites

Each experiment's focal parasite was quantified by qPCR, with quantities corrected for DNA concentration (i.e. ethanol precipitation of *O. lignaria* samples) where appropriate. Although we observed parasites by microscopy in preliminary trials with *C. bombi* and *O. lignaria*, we elected to use molecular quantification in the Factorial Cross-infection Experiment to enable unbiased and specific detection of all parasite morphotypes. For example, trypanosomatid infection with *L. passim* in *A. mellifera* is characterized by non-motile spheroid forms that adhere to the gut epithelium (Schwarz *et al*., 2015) and may not be detectable in feces or the supernatant of gut homogenate. Likewise, the intracellular stages of *N. ceranae* (Higes *et al*., 2007) would be undetectable by fecal spore counts. In addition, spheroid forms of trypanosomatids and spores of *N. ceranae* are both similar in size and shape to yeasts that co-occur in the bee gut. Compared to microscopic cell counts, we decided that molecular methods provided a relatively unbiased, reproducible and observer-independent means of parasite quantification.

*Crithidia bombi*, *C. mellificae* and *L. passim* were quantified as previously described (Ulrich *et al*., 2011; Palmer-Young *et al*., 2018*a*). Reactions were run in triplicate with primers for the *C. bombi* 18s rRNA gene [‘CriRTF2’ (GGCCACCCACGGGAATAT) and ‘CriRTR2’ (CAAAGCTTTCGCGTGAAGAAA)] (Ulrich *et al*., 2011). The nucleotide sequence targeted by the primers has 100% sequence identity for *C. bombi*, and a single mismatch in the reverse primer for *C. mellificae* and *L. passim*. However, amplification efficiency was >90% in all assays. Cycle times were converted to parasite cell quantities based on standard curves, derived from the DNA extract of cell cultures of the appropriate species to correct for any possible differences in amplification effectiveness across parasite species.

*Nosema ceranae* was quantified using primers specific to *N. ceranae* and excluding *N. apis* [NcF (AAGAGTGAGACCTATCAGCTAGTTG) and NcR (CCGTCTCTCAGGCTCCTTCTC)] (Bourgeois *et al*., 2010; Rubanov *et al*., 2019). Because we did not have access to cell cultures of this parasite, cycle times were converted to copy numbers based on a standard curve made by amplification of a purified plasmid (Rubanov *et al*., 2019). For full details of qPCR, see Supplementary Methods: Molecular quantification of infection.

### Statistical analyses

Data analyses were conducted using open-source statistical software R v3.6.1 for Windows (R Core Team, 2014); results were graphed with package *ggplot2* (Wickham, 2009) and the extension *cowplot* (Wilke, 2016).

*Crithidia bombi*–*Megachile experiment*. Within each host species, median parasite quantities were identical for bees (male *M. rotundata* and female *B. impatiens* controls) inoculated with parasites from feces and from centrifuged, resuspended gut extracts (*M. rotundata*: 17 500 cells per bee, *n* = 10 per method, *B. impatiens*: 22 500 cells per bee, *n* = 36 per method, Supplementary Fig. 1). Therefore, the results from the two inoculation methods were pooled. We compared prevalence of parasite detection between *M. rotundata* and the *B. impatiens* positive controls, pooled across trials with male and female *M. rotundata* to maximize statistical power. The proportion of bees with microscopically detectable parasites at 7 days post-inoculation was used as the response variable, and host species used as the predictor variable, in a binomial family generalized linear model (Bates *et al*., 2015). Significance of the predictor variable was evaluated using an *F*-test in package *car*, function ‘Anova’ (Fox and Weisberg, 2011). We acknowledge that this is an imperfect comparison due to pooling across trials – as necessitated by the asynchronous emergence of male and female *M. rotundata* – and provide these results for descriptive purposes only. Within each sex of *M. rotundata*, we tested the effect of bee size on parasite quantities. The count of parasites in 0.02 *μ*L gut homogenate was used as the response variable, and the intertegular distance (in mm) was used as the predictor variable. The model used a negative binomial family generalized linear model (Bliss and Fisher, 1953) implemented in R package *glmmTMB* (Brooks *et al*., 2017). We did not formally compare parasite quantities between *M. rotundata* males and females – which were inoculated with different methods on different dates – nor between *M. rotundata* and *B. impatiens*, which were inoculated with different numbers of parasite cells (6000 for *M. rotundata vs* 12 000 for *B. impatiens*) and differed dramatically in the variance of parasite quantities (see Fig. 2). We used a binomial family model to compare the proportion of deaths within 7 days between inoculated *M. rotundata* and *B. impatiens*, but could not directly assess the effects of inoculation on mortality due to absence of sham-inoculated controls.

*Factorial cross-infection experiment: infection*. Due to the different methods of inoculation and quantification used for different host–parasite combinations, and the unknown initial dose of parasites in bees allowed to feed *ad libitum* on the inoculum, we did not attempt formal statistical comparisons of parasite quantities across parasites or hosts. Instead, we present descriptive summaries of outcomes across the 16 host–parasite combinations. To infer successful infection (i.e. the ability of parasites to replicate in each host), we compared the amounts of parasites found at the end of the experiment with the quantity used for inoculation, with the following caveats: First, for the hosts *H. ligatus* and *O. lignaria*, the quantity of parasites inoculated could not be measured (see Methods: Inoculation above). Therefore, for these bees, we evaluated infection under the conservative assumption that these bees consumed the quantity of parasites found in the entire 200 *μ*L of inoculum. This is likely an overestimate – none of the bees consumed the entire inoculum and, except for accidental spills, we seldom had to replace the 1 mL sugar water tubes provided to *H. ligatus* and *O. lignaria* bees during the subsequent 7 days of the experiment, indicating daily consumption rates of <140 *μ*L. In addition, our estimates of parasite replication do not account for any cells excreted in feces, which may contain thousands of spores or cells per microlitre (Otterstatter and Thomson, 2006; Copley *et al*., 2012). Second, for the parasite *N. ceranae*, the quantity inoculated was measured as spore number but parasite quantities were measured in gene copy equivalents; for this parasite, we assumed a ratio of 10 gene copy equivalents per parasite cell (Bourgeois *et al*., 2010). For two host–parasite combinations – *H. ligatus* inoculated with *C. bombi* and with *C. mellificae* – the trial included *n* > 4 individuals each of males and females. Differences in parasite quantities by sex were assessed with a Wilcoxon signed-rank test using parasite quantities (number of parasite cell equivalents) as the response variable and host sex as the predictor variable.

*Factorial cross-infection experiment: mortality*. Due to the short time-span over which bees were monitored post-inoculation (8 days), our experiments were not ideally suited to assess the effects of parasite inoculation on mortality, and we generally had too few deaths to implement standard survival analyses. Instead, for each host–parasite combination, we compared the proportion of bees that died within 8 days between the parasite- and sham-inoculated treatment groups with a binomial family generalized linear model (Bates *et al*., 2015).

## Results

### *Crithidia bombi* infection in *M. rotundata*

The trial with *C. bombi* infection of *M. rotundata* and *B. impatiens* showed high prevalence of parasite detection in *M. rotundata*, which was statistically indistinguishable from that achieved in the primary host *B. impatiens* (Fig. 2). Pooled across the two trials, prevalence of detection did not differ between the two hosts (*M. rotundata*: mean 0.87 ± 0.03 s.e.; *B. impatiens*: mean 0.86 ± 0.07 s.e.; host effect: *F*_1, 123_ = 0.01, *P* = 0.91). Among *M. rotundata* with detectable *C. bombi*, extrapolated parasite quantities exceeded the 6000 cells used for inoculation in the majority of cases (males: 82%, females: 76%), indicating parasite replication. Median parasite quantities were similar for the trials with *M. rotundata* males and females (males: median 17 500 cells per bee; females: median 15 000 cells per bee; interquartile range: 5000 to 30 000 for cells per bee each sex). Compared to the inoculated dose of 6000 cells per bee, these median parasite quantities at 7 days post-inoculation represented increases in parasite cell numbers of 2.91-fold for males and 2.5-fold for females (Supplementary Fig. 1). There was a non-significant trend for larger bees to have higher parasite quantities in both males (*β* = 1.44 ± 0.78 s.e., *χ*^2^_1_ = 3.35, *P* = 0.067) and females (*β* = 2.36 ± 1.28 s.e., *χ*^2^_1_ = 3.39, *P* = 0.066).

Mortality was low in both *M. rotundata* and *B. impatiens*. In the trial with males, there was one death among 61 *M. rotundata* (1.6%), as compared to 1 death among 34 *B. impatiens* controls (2.9%). In the trial with females, there were three deaths among 33 *M. rotundata* (9.1%, all at 7 days post-inoculation) as compared to zero deaths among nine *B. impatiens* controls. Pooled across trials, probability of death within 7 days did not differ between *M. rotundata* and *B. impatiens* (*M. rotundata*: mean 0.04 ± 0.02 s.e., *B. impatiens*: mean 0.03 ± 0.03 s.e., *F*_1, 121_ = 0.04, *P* = 0.84). Because no sham-inoculated control treatment was included, we could not test the effects of exposure to *C. bombi* on *M. rotundata* mortality.

### Factorial cross-infection experiment: infectivity of four pathogens in four bee species

#### Infection

*Crithidia bombi*: In the factorial cross-infection experiment, *C. bombi* DNA was detected in abundance at 8 days post-inoculation in both *H. ligatus* and *O. lignaria*, but replication could only be confirmed in *O. lignaria*. In both alternative hosts, qPCR measures of parasite quantities after 8 days rivalled or eclipsed those found in the primary host *B. impatiens*. Parasite quantities exceeded 5000 cells in six of 17 *B. impatiens* (35%, all above the 10 000 cells used for inoculation), seven of 17 *H. ligatus* (41%) and nine of 25 *O. lignaria* (36%), indicating persistence of parasites in both alternative hosts (Fig. 3, Supplementary Fig. 2; see Supplementary Table 2 for full descriptive statistics). Compared to maximum parasite quantities in *B. impatiens* (1.42 × 10^5^ parasite cell equivalents), maximum quantity was similar in *H. ligatus* (7.67 × 10^4^ cell equivalents) and over 7-fold higher in *O. lignaria* (1.08 × 10^6^ cell equivalents). If parasite quantities at 8 days post-inoculation represent an asymptote or steady state (Otterstatter and Thomson, 2006), our results suggest that parasite carrying capacity in these alternative hosts is comparable to that in a primary host. Because we were unable to quantify the number of parasite cells inoculated per bee, we do not know the exact extent of parasite replication in these alternative hosts. Under the conservative assumption that every bee in the parasite treatment consumed the entire inoculum (2 × 10^5^ cells in 200 *μ*L), *C. bombi* replication could be inferred for zero *H. ligatus* and 3 of 25 (12%) of *O. lignaria*. However, such high parasite quantities (>2 × 10^5^ cell equivalents) were not observed in the primary host *B. impatiens* either, and might exceed the carrying capacity of the smaller-bodied *H. ligatus*.

Our molecular quantification of parasites did not assess their viability. However, we observed red fecal stains in the rearing cups of all species within 24 h of inoculation, suggesting that the detection of parasites 8 days post-infection is unlikely to reflect passive retention of dead initially inoculated cells (or their nucleotides) in the gut. Any parasites that persisted in the gut throughout the experiment must have been sufficiently alive to actively maintain their positions in the gut, e.g. by swimming or embedding in the epithelium (Gorbunov, 1996; Koch *et al*., 2019), whether or not they were replicating. Minimal parasite quantities were found in *A. mellifera* (maximum 96 parasite cell equivalents, i.e. <2% of the quantity inoculated).

*Crithidia mellificae*: *Crithidia mellificae* parasite quantities at 8 days post-inoculation were highest in *H. ligatus* and *O. lignaria*, rather than in the primary host *A. mellifera*. In *A. mellifera* (median 77.6, max 1.10 × 10^6^ parasite cell equivalents), 5 of 18 bees had more than the 5000 parasites cells inoculated and three had parasite quantities >10^5^ cell equivalents. In *H. ligatus*, however, infection was detected in all 15 parasite-inoculated samples, with 13 of 15 bees having more than 10^5^ parasite cell equivalents (Fig. 3). Median parasite quantity (2.24 × 10^6^ cell equivalents) was over four orders of magnitude higher than in *A. mellifera*, and maximum quantity (7.56 × 10^6^) was nearly 7-fold higher. In *O. lignaria*, 5 of 11 bees had more than 10^5^ parasite cell equivalents. Median parasite quantity (8310 cell equivalents per bee) was over 100-fold higher than in *A. mellifera*, while maximum quantity (3.56 × 10^6^ cell equivalents) was over 3-fold higher. The fact that parasite quantities in both alternative hosts approached or exceeded this level – far beyond the observed intestinal transit time for the inoculum – indicates that both of these alternative hosts provide suitable habitats for *C. mellificae*, with carrying capacities not inferior to that of a primary host. As with *C. bombi*, our inability to measure quantities inoculated does not allow precise calculation of net parasite replication in *H. ligatus* and *O. lignaria* over the 8 days post-inoculation. However, even under the conservative assumption that each bee consumed the entire 200 *μ*L of inoculum (2 × 10^5^ cells), our results still provide evidence of *C. mellificae* replication in 87% of *H. ligatus* and 45% of *O. lignaria*. These proportions exceed the 28% of *A. mellifera* – the primary host – with confirmed parasite replication. In contrast, no evidence of infection was found in *B. impatiens*, with none of the infections exceeding the 10^4^ cells used for inoculation, and only one sample with >100 parasite cell equivalents (i.e. 1% of the quantity inoculated).

*Lotmaria passim*: Inoculation with the *A. mellifera*-associated *L. passim* was generally unsuccessful in *A. mellifera*, with even lower parasite quantities found in *B. impatiens*, *H. ligatus* and *O. lignaria*. Among inoculated *A. mellifera*, only 2 of 21 (22%) parasite-inoculated bees harboured more parasites at dissection than the 5000 cells used for inoculation, with a maximum of 3.73 × 10^5^ parasite cell equivalents (Fig. 3). The next-highest quantity (1050 parasite cell equivalents in *H. ligatus*) was <1% of the maximal quantity in *A. mellifera*; this was the only non-*A. mellifera* sample with more than 1000 parasite cell equivalents. Although we cannot rule out that failure of infection in *H. ligatus* and *O. lignaria* reflects poor consumption of the inoculum, we successfully infected these alternative hosts with *C. bombi* and/or *C. mellificae* under the same conditions. This suggests that the absence of infection with *L. passim* reflected incompatibility with these hosts, and was not solely due to low quantities inoculated.

*Nosema ceranae*: Like *L. passim*, the *A. mellifera-*associated *N. ceranae* achieved little cross-infection in any of the candidate alternative hosts, but was also inconsistently infectious in the primary *A. mellifera* host. *Nosema ceranae* detections were dominated by 7 of the 19 samples of *A. mellifera*, with two high outliers reaching a maximum of 6.45 × 10^7^ gene copy equivalents (Fig. 3). However, median parasite quantity was nearly two thousand-fold lower (3570 gene copies) – lower than the maximum quantity among sham-inoculated *A. mellifera* controls (14 100 gene copies). Assuming 10 copies of the target gene per parasite genome (Bourgeois *et al*., 2010), we found evidence of parasite replication (i.e. quantities >5000 spores per bee used for inoculation) in 28% of *A. mellifera*. Parasite quantities were even lower among the other three host species. Together, these candidate hosts accounted for only four detections above 1000 copies – all in *O. lignaria* – with a maximum quantity (14 200 copies, or ~1420 parasites) similar to maximum infection in sham-inoculated *A. mellifera* (Supplementary Fig. 2). As with the trypanosomatid parasites, we cannot calculate the extent of parasite replication in *H. ligatus* and *O. lignaria* due to unknown quantities inoculated; however, our findings give no indication that *N. ceranae* infects these alternative hosts.

*Trypanosomatids detected in sham-inoculated H. ligatus controls*: Although sample sizes were smaller for some negative control (i.e. sham-inoculated) groups, none of the sham-inoculated bees had high numbers of parasites (Fig. 3). Moreover, the same primers were used for all trypanosomatids, which means that there is built-in redundancy of the negative controls used for experiments with the three trypanosomatid parasites (*C. bombi*, *C. mellificae* and *L. passim*). If this redundancy is considered, each host bee species has a minimum of *n* = 24 negative controls for pre-existing trypanosomatid infection (Fig. 3).

However, in two of the three experiments that tested trypanosomatid infection, our non-specific trypanosomatid qPCR primers detected high prevalence – but low quantities – of trypanosomatids in wild-collected *H. ligatus*. For the week of experiments with *C. bombi*, trace amounts of trypanosomatids were found among all nine sham-inoculated *H. ligatus* (median 2.4, max 10 parasite cell equivalents). In comparison, trypanosomatids were detected in 0 of 9 sham-inoculated *A. mellifera*, 3 of 11 *B. impatiens* and 2 of 17 *O. lignaria* (Supplementary Fig. 2). For experiments with *C. mellificae*, all six of the sham-inoculated *H. ligatus* had detectable trypanosomatids (median 46.5, maximum 339 parasite cell equivalents). These levels were again high compared to *A. mellifera* (6 of 11 bees with detectable trypanosomatids, median infection 2.6 parasite cell equivalents), *B. impatiens* (1 of 10 bees) and *O. lignaria* (0 of 2 bees). In the week of experiments with *L. passim*, prevalence of trypanosomatid detection was lower (one detection among nine sham-inoculated bees), but *H. ligatus* accounted for the highest quantity among sham-inoculated bees (821 parasite cell equivalents); this was 100-fold higher than the next-highest quantity in the sham treatment (8.3 cell equivalents in *O. lignaria*, Supplementary Fig. 2). The consistently high detection prevalence and quantity of trypanosomatids found in sham-inoculated *H. ligatus* (relative to other sham-inoculated hosts) suggests that these low-level detections are unlikely to result from experimental error. Instead, they suggest pre-existing but persistent trypanosomatids – of unknown source and identity – in the guts of wild-collected bees and their local population.

### Mortality

Binomial models did not reveal elevated mortality due to parasite inoculation in any of the alternative hosts (Supplementary Fig. 3 and Supplementary Results).

## Discussion

Our findings show evidence for establishment and persistence of bee-infective intestinal trypanosomatids outside of the primary host, particularly for *Bombus*-derived *C. bombi* and the multi-host parasite *C. mellificae*. Due to the less controlled nature of inoculations, our results with *H. ligatus* and *O. lignaria* are less robust than those with *M. rotundata*. However, our findings still provide evidence of *C. bombi* replication in *O. lignaria* and *C. mellificae* replication in both *O. lignaria* and *H. ligatus*, even under maximally conservative assumptions. Even for cases where parasite replication could not be confirmed, results show the persistence of parasites far beyond the duration expected based on the transit time of the inoculum, with parasite quantities at 8 days post-inoculation comparable to those found in the primary host. These findings are consistent with the generally low host specificity of extracellular, monoxenous trypanosomatids of insects (Kozminsky *et al*., 2015), and substantiate the potential for pathogen spillover from managed *Apis* and *Bombus* to co-occurring pollinator species in other genera. In comparison, the trypanosomatid *L. passim* and the microsporidian *N. ceranae* showed little cross-infection, although they also resulted in inconsistent infection of the primary host *A. mellifera*. The consequences of cross-infection with trypanosomatids for alternative hosts – and the ecosystem services that they render – remain unknown.

The trypanosomatids *C. bombi* and *C. mellificae* showed the strongest potential for cross-infection. Parasite quantities in parasite-inoculated bees far exceeded those in sham-inoculated controls for seven of the nine host–parasite combinations tested, indicating persistence of parasites post-inoculation (Figs 2 and 3). Our results include the first demonstration of experimental *C. bombi* infection outside of *Bombus* spp. – in two novel hosts (*M. rotundata* and *O. lignaria*) – and the first reports of *C. mellificae* infection in *O. lignaria* and *H. ligatus*. For both parasites, parasite quantities and prevalence of detection in alternative hosts rivalled or exceeded that found in primary hosts. Our inability to measure the parasite quantities inoculated in *H. ligatus* and *O. lignaria* makes it impossible to estimate parasite replication precisely in these hosts. However, the high absolute parasite quantities found 8 days post-inoculation strongly suggest that that both *H. ligatus* and *O. lignaria* are competent hosts for *C. bombi* and/or *C. mellificae*; more closely controlled inoculations of *H. ligatus* with *C. bombi* are required to evaluate parasite replication.

In addition to the primary host *B. impatiens*, *C. bombi* infected the two megachilids (*M. rotundata* and *O. lignaria*); although *C. bombi* was also found in *H. ligatus*, we could not confirm net parasite replication in this species, as none of the inoculated bees had more than the 200 000 cells offered in the inoculum. Parasites were most consistently detected in *M. rotundata* (86% of males and 88% of females), as compared to 95 and 67% in *B. impatiens* controls in the corresponding trials (Fig. 2). Moreover, estimated parasite quantities at 7 days post-inoculation were consistently higher than the quantity inoculated, which indicates successful *C. bombi* replication in *M. rotundata*. Our counts on experimental bees likely represent conservative estimates of parasite quantities, because they ignore non-motile trypanosomatid forms [variously called ‘amastigotes’ (Logan *et al*., 2005) and ‘spheroids’ (Schwarz *et al*., 2015)] that failed to swim into the supernatant of settled gut homogenate. Although we cannot entirely rule out that some of these bees had pre-existing infection, the fact that experimental bees were raised in the lab – without exposure to infected *Bombus* or flowers – makes prior infection highly unlikely. In holometabolous Hymenoptera with separate larval, pupal and adult stages – including *Bombus* spp. and *Megachile* spp. – the gut undergoes extensive remodelling during metamorphosis, including complete excretion of its contents (Engel and Moran, 2013). This remodelling and excretion would eliminate any trypanosomatids in the hindgut. Accordingly, newly emerged adult *Bombus* are *Crithidia-*free (Otterstatter and Thomson, 2006). Moreover, our microscopic examination of 50 sham-inoculated *M. rotundata* from the same supplier and year found no evidence of trypanosomatids (Figueroa *et al*., in preparation).

The samples with the greatest *C. bombi* quantities in the factorial cross-infection experiment (Fig. 3) were from *O. lignaria* – not the primary host *B. impatiens*. Our results with *O. lignaria* are probably an underestimate of true parasite quantities due to the poor DNA yield from gut samples, which required ethanol precipitation to raise host DNA concentrations to PCR-detectable levels (see Methods: DNA extraction and quality control). In support of extraction-limited parasite detection, the two samples with the highest parasite quantities were both from samples that did not require precipitation. In *B. impatiens* and *B. terrestris*, *C. bombi* prevalence can exceed 50–80% of workers (Shykoff and Schmid-Hempel, 1991; Schmid-Hempel, 2001; Gillespie, 2010). Our results indicate that *C. bombi* persists with comparable frequency and at comparable amounts in the alternative hosts – *M. rotundata* and *O. lignaria* – and the primary host *B. impatiens*. Given that all of these host species are generalists and could therefore exchange parasites at shared flowers, *C. bombi* prevalence could be similarly high in megachilid populations that are sympatric with infected *Bombus* spp., with possible transmission among Megachilid populations as well. Like *C. bombi*, *C. mellificae* was infectious in *O. lignaria*, but also in *H. ligatus*, with maximum parasite quantities in each of these two hosts exceeding that of the primary host, *A. mellifera* (Fig. 3). These parasite quantities are particularly remarkable given the small body size of *H. ligatus* (Stone and Willmer, 1989).

The ability of *Crithidia* spp. and other monoxenous trypanosomatids to complete their life cycles within the gut tract (Wallace, 1966) may facilitate their ability to cross-infect alternative hosts with similar diets or gut physiology, as might be expected among different species of nectar- and pollen-consuming bees. For example, the amino acid composition of many floral nectars (Carter *et al*., 2006) and pollens is dominated by proline (De Simone *et al*., 1980; Mondal *et al*., 1998; Yang *et al*., 2013). This amino acid can be used as a carbon source by insect gut trypanosomatids (Bringaud *et al*., 2006), and may facilitate colonization of diverse bee hosts with proline-rich diets. The gut-specific nature of trypanosomatid infection may also enable avoidance of the host immune system, such as phagocytes and antimicrobial peptides, that target trypanosomatids in the haemolymph (Boulanger *et al*., 2001). Although infection with *C. bombi* often upregulates transcription of antimicrobial peptide genes in *B. terrestris* (Riddell *et al*., 2009; Barribeau and Schmid-Hempel, 2013), successful parasite strains elicit relatively little immune gene activity (Barribeau *et al*., 2014). Similarly, RNA sequencing of *L. passim-*inoculated *A. mellifera* revealed remarkably little alteration of the host transcriptome (Liu *et al*., 2019).

A deeper understanding of the relative suitability of different hosts for parasites could be achieved by comparing parasite morphologies and patterns of colonization in primary *vs* alternative host species. For example, both *C. mellificae* and *L. passim* form a layer of spheroid cells that line the hindgut and rectum of *A. mellifera*, with free-swimming promastigote forms found in the lumen (Schwarz *et al*., 2015). *Crithidia bombi* also exhibits site-specific colonization and morphology in the primary host *B. terrestris* (Koch *et al*., 2019). Elongated (choanomastigote) morphotypes line the ileal epithelium, where they are anchored by their flagella (Koch *et al*., 2019). However, parasites may also be found swimming freely in the ileal gut lumen, and accompanied by putatively transmissive spheroid forms in the rectum (Gorbunov, 1996). Whether these interactions with the gut epithelium and site-specific morphologies are also observed in alternative hosts requires further study. Such interactions could affect the activation of host immunity, parasite transmission and host morbidity due to, e.g. competition for nutrients, water balance and damage to gut tissue (Schaub, 1994). For example, inoculation with *C. mellificae* can elevate mortality of *O. cornuta*, at least in males (Strobl *et al*., 2019). Otherwise, the effects of parasites on alternative hosts – and the biotic and abiotic factors that affect parasite establishment and host resistance and tolerance – remain largely unknown, but are currently under investigation (Laura Figueroa and Scott McArt, unpublished data).

Counter to our hypothesis that the closely related *B. impatiens* and *A. mellifera* would share parasites, the only species with negligible persistence of *C. bombi* was *A. mellifera*; there was likewise no infection of the *A. mellifera*-derived *L. passim* in *B. impatiens* (Fig. 3). We hypothesize that two factors – gut microbiota and temperature – may confer resistance to non-specialist parasites in these social bee species. *Bombus impatiens* and *A. mellifera* are both corbiculate (‘pollen-basket’) bees within the family Apidae, and both harbour a socially transmitted, phylogenetically similar gut microbiota (Kwong *et al*., 2017) that is a key mediator of trypanosomatid infection in the *Bombus* spp./*Crithidia* spp. system (Koch and Schmid-Hempel, 2011, 2012; Mockler *et al*., 2018; Palmer-Young *et al*., 2019*a*, 2019*b*). In contrast, solitary bees – as well as the facultatively eusocial Halictids – lack the socially transmitted core gut microbiota that is a feature of *B. impatiens* and *A. mellifera* (McFrederick *et al*., 2012, 2014, 2016; Kwong *et al*., 2017). Instead, their guts are colonized by environmental bacteria and other microbes acquired at flowers and in nests (McFrederick *et al*., 2016). This lack of socially reinforced, antiparasitic gut bacteria could elevate susceptibility to trypanosomatids. In contrast, the presence of inhibitory, gut-specialist microbiota in a suboptimal host species could limit infection of *C. bombi* in *A. mellifera* and of *L. passim* in *B. impatiens*, despite the physiological similarity of these two host species. As an additional caveat, susceptibility to trypanosomatid and microsporidian infection can differ dramatically across colonies of the same species (Koch and Schmid-Hempel, 2012; Chaimanee *et al*., 2013; Barribeau *et al*., 2014). Hence, we cannot exclude the potential for infection outside of the single *A. mellifera* and seven *B. impatiens* colonies tested here.

Another possible explanation for the lack of *C. bombi* infection in *A. mellifera* is the high incubation temperature used for the *A. mellifera* in our experiments. Whereas all other bee species were reared at 30°C in individual containers, *A. mellifera* bees were reared in groups at 35°C. We used the higher 35°C temperature for *A. mellifera* to minimize post-emergence changes in temperature [brood temperatures are regulated at ~34.5°C (Williams *et al*., 2013)] and optimize survival (Clinch and Faulke, 1977). However, 30°C conditions for *A. mellifera* adults have been used successfully by other authors (Forsgren and Fries, 2010; Williams *et al*., 2013), and future cross-infection experiments should ideally apply the same temperature conditions for all hosts. For example, the 28–32°C temperature range is ideal for growth of *C. bombi*, whereas higher temperatures inhibited *in vitro* parasite growth, potentiated the antagonistic effects of gut symbionts (Palmer-Young *et al*., 2018*b*), and reduced infection prevalence and intensity in *B. impatiens* (Palmer-Young *et al*., 2019*a*, 2019*b*). The only study to date that found temporary persistence of *C. bombi* in *A. mellifera* used a lower rearing temperature of 30°C (Ruiz-González and Brown, 2006), which is within the range of peak *C. bombi* growth rate *in vitro* (Palmer-Young *et al*., 2018*b*) and allowed substantial infection in *B. impatiens* (Palmer-Young *et al*., 2019*a*, 2019*b*).

Temperature affects susceptibility of *A. mellifera* to fungal (including *N. ceranae*) and viral infections (James, 2005; Martín-Hernández *et al*., 2009; Dalmon *et al*., 2019) and is generally important in host–parasite interactions (Molnár *et al*., 2017; Kirk *et al*., 2018), including the effects of emerging infectious diseases (Raffel *et al*., 2013). Whereas the social lifestyles of *Apis* and *Bombus* spp. bees allow maintenance of consistently high nest temperatures (Esch, 1960; Heinrich, 1972, 1974) that could constrain establishment of environmental microbes (Casadevall, 2016), the relatively low and variable temperatures experienced by small solitary bees in the wild could increase their susceptibility to trypanosomatid infection. Studies of these and additional host species could discern the importance of physiochemical properties of the gut lumen – such as microbiota, pH, temperature and pollen type and availability (Koch and Schmid-Hempel, 2011; Conroy *et al*., 2016; Giacomini *et al*., 2018; Palmer-Young *et al*., 2018*b*, 2019*a*, 2019*b*) – for establishment of infection.

Unlike *C. bombi* and *C. mellificae*, neither *L. passim* nor *N. ceranae* showed high infectivity in alternative hosts, but the relatively low infectivity in the primary host *A. mellifera* makes it difficult to rule out the cross-infectivity of these parasites. In the case of *L. passim*, we are unaware of any studies that experimentally tested for infectivity of *L. passim* outside of *A. mellifera* (Schwarz *et al*., 2015). Although a previous experiment tested the infectivity of *A. mellifera*-derived gut trypanosomatids – which could have been *L. passim* (Ravoet *et al*., 2015) – in *B. terrestris*, the concentration of parasites in the inoculum was extremely low [<0.1 cells per bee *vs* 10 000 cells per bee for *Bombus*/*Crithidia* experiments (Ruiz-González and Brown, 2006)], resulting in a weak test of cross-infectivity. Without results from a concentrated, highly infectious inoculum, we cannot exclude the ability of *L. passim* to infect non-*Apis* hosts. Likewise, we cannot rule out that higher quantities inoculated [e.g. 10^5^ cells (Liu *et al*., 2020)] or repeated exposures to infectious parasites would result in greater infection; these factors should be tested in future studies.

In contrast to the narrow documented host range of *L. passim*, infectivity and/or virulence of *N. ceranae* has been demonstrated both within (Chaimanee *et al*., 2013) and outside of *Apis*, including in stingless bees (Purkiss and Lach, 2019) and *B. terrestris* (Graystock *et al*., 2013; Fürst *et al*., 2014). However, *N. ceranae* infection failed to establish in a subsequent experiment with *B. terrestris* reported by one of the same labs (Piiroinen *et al*., 2016), despite a 130 000 spores per bee inoculation treatment that was 20-fold higher than the 6500 spores successfully used previously (Graystock *et al*., 2013). A recent comprehensive series of experiments in *B. terrestris* supported this negative result (Gisder *et al*., 2020). Experimental inoculations with *N. ceranae* were also conducted with *O. bicornis* (Müller *et al*., 2019), but results are ambiguous with respect to infectivity. Although parasite DNA was detected in inoculated hosts, infection was evaluated only by qualitative PCR of the entire body – not by quantitative PCR of the gut as performed in our experiments – making it difficult to gauge the ability of parasites to replicate in the gut itself. Hence, the consistency of *N. ceranae* infectivity within *B. terrestris* and the extent of the parasite's infectivity in other bee species remain unclear. As with *L. passim*, *N. ceranae* cross-infectivity could be more conclusively tested with an inoculum that is strongly and consistently infectious in the original *A. mellifera* host. This could involve inoculation with greater quantities of parasites [e.g. 3 × 10^5^ spores per bee (Purkiss and Lach, 2019)] or allowing more time for development of infection [e.g. 10–14 days (Forsgren and Fries, 2010)]. Although the lack of cross-infection by *N. ceranae* in our experiments is consistent with our hypothesis of higher host specificity among intracellular microsporidia than extracellular trypanosomatids, the low success of *N. ceranae* in *A. mellifera* suggests that further research is needed to evaluate this idea.

Although in some insect/parasite systems, the potential host range of parasites may exceed the number of species in which they are actually observed (Perlman and Jaenike, 2003), the broad and human-facilitated distribution of managed bees, their extensive foraging range and polylectic food preferences and their season-long activity all likely enhance the potential for parasite transmission to susceptible hosts. *Apis mellifera*, *B. impatiens*, *M. rotundata* and *O. lignaria* all forage from multiple floral species and are deliberately distributed for crop pollination (Velthuis and van Doorn, 2006; Pitts-Singer and Cane, 2011; Boyle and Pitts-Singer, 2019). The unmanaged *H. ligatus* also has a broad host range, ranging from the Nearctic to the tropics, and visits a variety of flowers (Packer, 1986; Packer and Knerer, 1987). Moreover, the diel activity patterns of small or solitary bees such as *M. rotundata*, *O. lignaria* and *H. ligatus* could elevate the risk of parasite acquisition. Small solitary bees lack the thermoregulatory capabilities of *Bombus* spp. (Stone and Willmer, 1989). As a result, they often forage later in the day on flowers previously visited by *Bombus* (Heinrich, 1976*b*, 2004). Florally transmitted trypanosomatids can remain motile for up to 2 h after deposition on flowers (Figueroa *et al*., 2019), and trypanosomatid prevalence in the reservoir species *B. terrestris* species tends to increase over the season in Europe (Schmid-Hempel, 2001). Given that *Bombus* spp. in temperate climates are active throughout daylight hours during the growing season (Heinrich, 2004), it is therefore plausible that solitary bees would be exposed to parasites deposited by infected bumble bees earlier in the day. Although smaller *A. mellifera* lack some of the individual thermoregulatory capacity of *Bombus* spp., the high densities of bees that may occur near apiaries and their wide-ranging foraging habits could provide a continuous inoculation of shared flowers with potentially infectious parasites. On the other hand, the seasonally restricted early-spring activity of some solitary bees – which can occur before the seasonal buildup of *Bombus* spp. workers (Heinrich, 1976*b*) – may reduce the chances of transmission to these early-season species. Field studies of diverse bee populations are needed to evaluate these hypotheses.

In *H. ligatus*, where life history mirrors that of *Bombus* spp., the successful inoculation of *C. mellificae* and evidence of low-level pre-existing trypanosomatid prevalence in wild bees are suggestive of future study on the dynamics and effects of infection. As in *Bombus*, the *H. ligatus* colony cycle includes spring nest initiation by overwintered queens, followed by development of eusocial colonies that produce the next generation of foundress queens (Packer, 1986). As a result, many of the effects of *Crithidia* spp. infection in *Bombus* spp. (Sadd and Barribeau, 2013) may also occur in *H. ligatus*. For example, the overwintering stage is the period where trypanosomatid infection is most virulent in *B. terrestris* (Brown *et al*., 2003; Fauser *et al*., 2017), and could elevate mortality of hibernating *H. ligatus* as well. Trypanosomatid infection in *H. ligatus* could also have similar within-colony transmission dynamics (Otterstatter and Thomson, 2007) and negative effects on nest establishment (Brown *et al*., 2003). Some sham-inoculated *H. ligatus* harboured low-intensity, presumably pre-existing trypanosomatid infections. Whether these were due to spillover from local *A. mellifera* (no *Bombus* spp. were present in the region of collection) or a Halictid-specific trypanosomatid warrants further investigation, with the possibility to reveal host-specific parasite strains or novel trypanosomatid species (Tripodi *et al*., 2018). We did not evaluate differences in infectivity among the individual parasite strains used for our inoculations. However, strain-specific compatibilities with alternative host species – similar to the strong genotype × genotype interactions observed in the *B. terrestris*/*C. bombi* system (Sadd and Barribeau, 2013; Barribeau *et al*., 2014) – could shape patterns of interspecific transmission and parasite population structure (Ruiz-González *et al*., 2012).

## Conclusion

Epidemiological theory postulates that infectious diseases with effective spatial transmission from reservoir hosts into smaller populations have the greatest potential to cause species extinction (Castro and Bolker, 2005). Our study documents the ability of parasites associated with highly mobile, socially nesting managed bees to infect novel hosts in different taxonomic families, defining the potential for pathogen spillover into populations of alternative hosts. Floral transmission-mediated pathogen spillover between pollinator populations has been repeatedly demonstrated at scales ranging from cages to continents (Otterstatter and Thomson, 2008; Schmid-Hempel *et al*., 2014; Graystock *et al*., 2015, 2016; Alger *et al*., 2019). Thus, infection of the bees studied here – and possibly other, equally susceptible species – may contribute to or amplify the effects of managed social bees on other bee species (Thomson, 2004; Mallinger *et al*., 2017; Wojcik *et al*., 2018). Coexistence with infected *A. mellifera* and *Bombus* spp. in low-quality environments could present simultaneous exposure to parasites, competition for food and exposure to pesticides (Goulson *et al*., 2015; Rundlöf *et al*., 2015). Although long-term mortality and sublethal effects of infection in alternative hosts remains to be determined, infection in *B. impatiens* and *B. terrestris* can reduce tolerance to other stressors, such as food deprivation (Brown *et al*., 2000, 2003) or nectar chemicals (Palmer-Young *et al*., 2017) and reduce foraging efficiency (Otterstatter *et al*., 2005; Gegear *et al*., 2006), thereby exacerbating the effects of food scarcity. The combined effects of these potential stress factors may ultimately promote declines in non-*Apis* bee populations (Williams *et al*., 2009; Vanbergen and Insect Pollinators Initiative, 2013) and the pollination services they provide (Klein *et al*., 2007; Winfree *et al*., 2008; Garibaldi *et al*., 2013).

## Data Availability

All raw data are available in the Supplementary materials.

## Supplementary material

For supplementary material accompanying this paper visit https://doi.org/10.1017/S0031182020001018. click here to view supplementary material
