# Supplementary material for: Cross-infectivity of honey and bumble bee-associated parasites across three bee families
Source: Parasitology. 2020 Jun 18;147(12):1290–304. doi: 10.1017/S0031182020001018 (PMC7477370; doi:10.1017/S0031182020001018)
Supplement: Supplementary file 1 [file S0031182020001018sup.zip › S0031182020001018sup002.pdf]

Supplementary information for: Cross-infectivity of honey and bumble bee-associated parasites across three bee families

Running title: Cross-infection of bee parasites

Authors

Lyna Ngor [1], Evan C Palmer-Young [1*&], Rodrigo Burciaga Nevarez [1], Kaleigh A Russell [1], Laura Leger [1], Sara June Giacomini [2], Mario S Pinilla-Gallego [2], Rebecca E Irwin [2], Quinn S McFrederick [1]

Affiliations

[1] Department of Entomology, University of California Riverside, Riverside, CA, USA

[2] Department of Applied Ecology, North Carolina State University, Raleigh, NC, USA

*Corresponding author: ecp52@cornell.edu

[&] Current address: USDA-ARS Carl Hayden Bee Research Laboratory, Tucson, AZ, USA

CONTENTS

Supplementary Figures 3
Supplementary Results 8
  Effects of host sex on infection intensity 8
  Effects of parasite inoculations on mortality 8
Supplementary Tables 10
Supplementary Methods 12
  DNA cleanup and concentration 12
  Molecular quantification of infection 13
Key to Supplementary data 15
  Crithidia bombi-Megachile experiment data 15
  Factorial cross-infection experiment: Infection 16
  Factorial cross-infection experiment: Mortality 17
References 18

SUPPLEMENTARY FIGURES

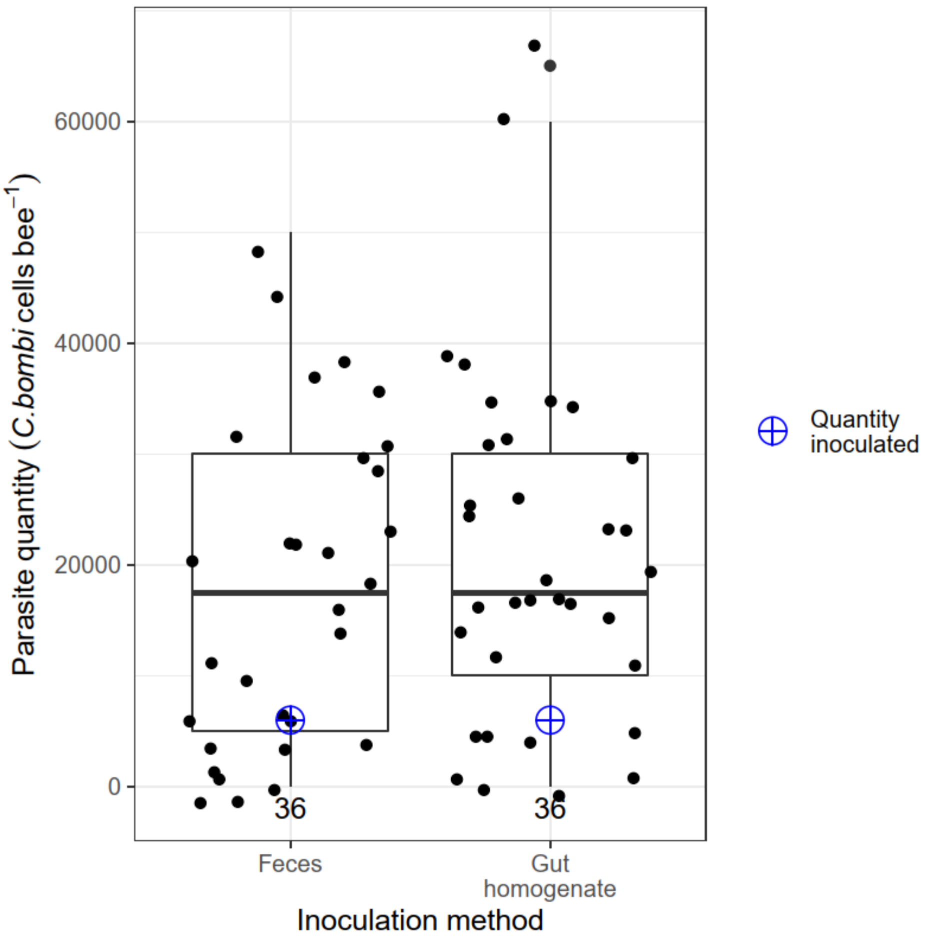

Supplementary Figure 1. Comparison of *Crithidia bombi* infection in *Megachile rotundata* males inoculated by two methods: parasites from diluted *Bombus* feces and from gut homogenate**.** X-axis shows inoculation method, Y-axis shows parasite quantities at 7 d post-inoculation relative to quantity inoculated (6000 cells bee^{-1}, blue hatched circles), extrapolated from hemocytometer parasite cell counts of gut homogenate. Points have been randomly offset from one another to avoid overplotting. Numbers along X-axis indicate sample sizes.

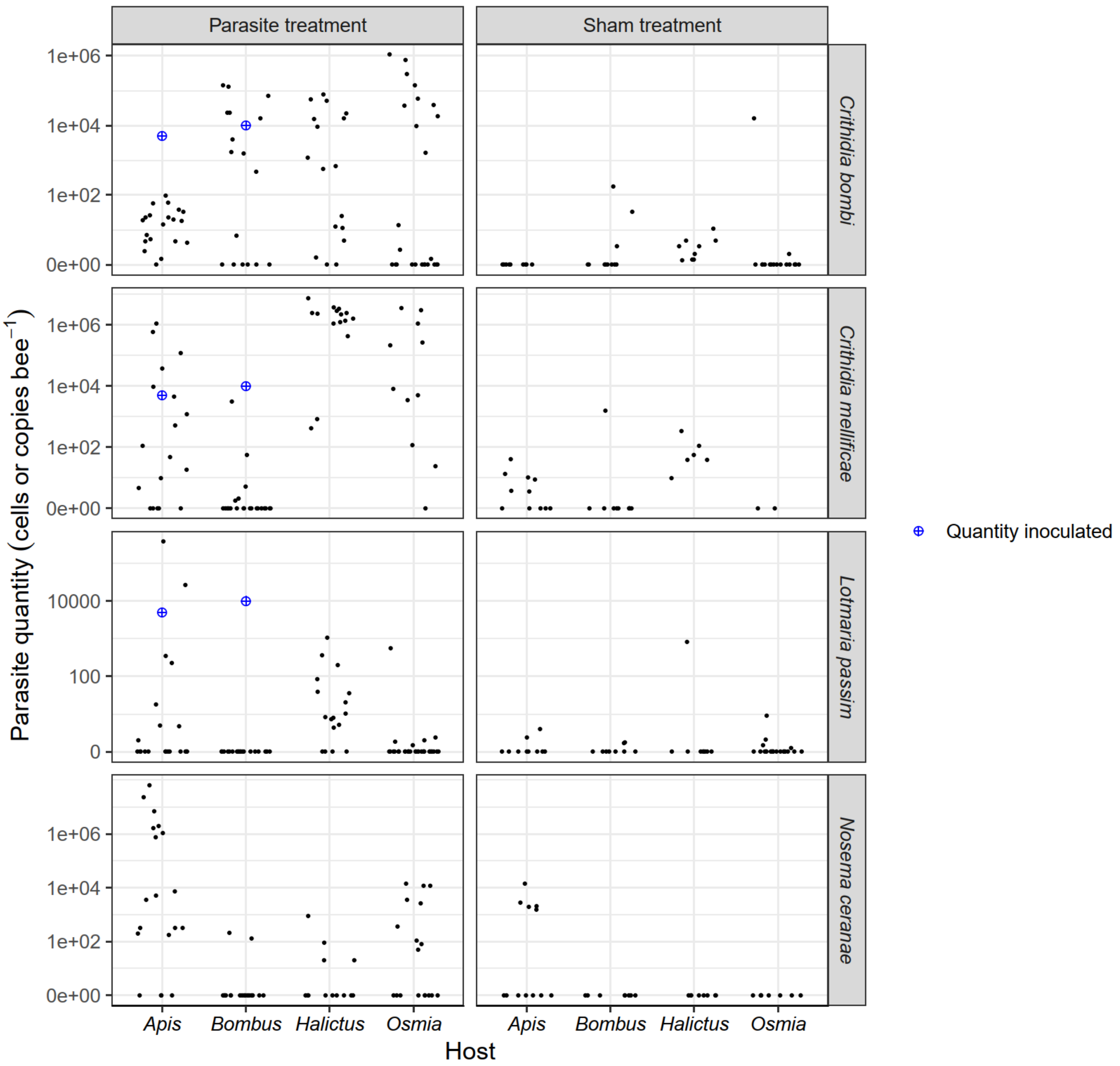

Supplementary Figure 2. Log-scale infectivity of four parasites across bee species of three families: *Apis mellifera* (Apidae)*, Bombus impatiens* (Apidae)*, Halictus ligatus* (Halictidae)*,* and *Osmia lignaria* (Megachilidae)*.* Points show estimated infection of each individual based on qPCR, randomly offset to the left and right to avoid overplotting. Y-axis for each parasite corresponds to standards used in qPCR (cell equivalents for the trypanosomatids *Crithidia bombi, Crithidia mellificae,* and *Lotmaria passim*; plasmid copy equivalents for the microsporidian *Nosema ceranae*). Samples with Cq > 40 are plotted as zeroes. Hatched circles indicate the number of cells with which bees were inoculated (10,000 for

Bombus, 5,000 for *Apis,* not quantified for *Halictus* or *Osmia.* The latter two hosts were offered 200,000 cells in 200 µL of inoculum).

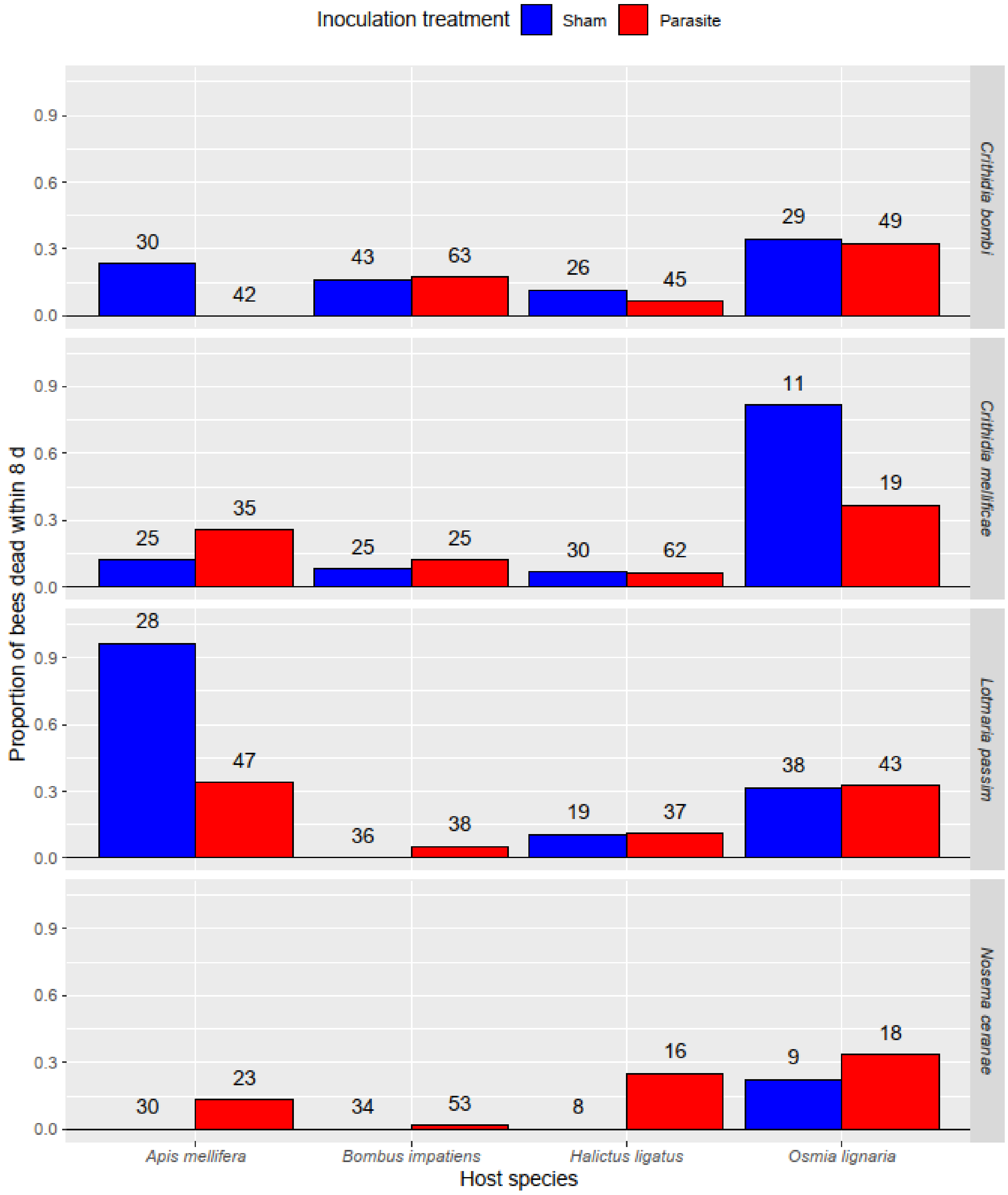

Supplementary Figure 3. Probability of death within 8 d post-inoculation. Proportion of deaths (y-axis) within 8 d of inoculation for four bee species (columns) inoculated with four parasite species (rows).

Blue bars indicate sham-inoculated bees; red bars indicate parasite-inoculated bees. The number on top of each bar shows the total number of bees used in experiment. The high mortality of *Apis mellifera* in the *Lotmaria passim* experiment coincided with a mid-experiment change of incubator settings.

SUPPLEMENTARY RESULTS

Effects of host sex on infection intensity

We found significant effects of host sex on rank-based infection intensity in one of four testable cases (N > 4 for each sex). In *H. ligatus*, *C. bombi* was significantly higher in males than females (W = 9, P = 0.031); infection intensity exceeded 10,000 parasite cell equivalents in 4 of 5 males but only 2 of 12 females. *Crithidia mellificae* infection intensity did not differ by sex in *H. ligatus* (W = 39, P = 0.18) or in *O. lignaria* (W = 20, P = 0.43), nor did *N. ceranae* infection differ by sex in *O. lignaria* (W = 34, P = 0.92). Note that small and unbalanced sample sizes (Supplementary Table S1) limited the statistical power of most comparisons, and lack of significant differences in our analyses does not rule out sex-specific differences in susceptibility to infection. In addition, we cannot rule out that the effects of sex with *H. ligatus* in our experiments reflect differential consumption of the inoculum rather than differential susceptibility to *C. bombi.*

Effects of parasite inoculations on mortality

Binomial models did not reveal elevated mortality due to parasite inoculation in any of the alternative hosts (Supplementary Figure 3). The strongest effects of inoculation treatment were found in *A. mellifera.* Probability of *A. mellifera* death was lower among sham-inoculated bees in the experiment with *L. passim* (χ^2_1 = 33.4, P < 0.001). However, this experiment was anomalous in that a surge of deaths (including 27 of 28 sham-inoculated bees) coincided with a mid-experiment change of incubator settings. Probability of *A. mellifera* death was also lower among sham-inoculated bees inoculated *with C. bombi* (χ^2_1 = 13.63, P < 0.001). In contrast, probability of death tended to be higher among parasite-inoculated bees in the experiment *with N.* ceranae (χ^2_1 = 5.25, P = 0.022), but this result is not significant at a Bonferroni-adjusted critical p-value (i.e., P = 0.0031) to account for the 16 multiple comparisons in the Factorial Cross-infection Experiment. All analyses on *A. mellifera* are presented for descriptive

purposes only, because all bees of a given treatment were reared within the same cage. This design confounded the effects of treatment with those of cage, and led to non-independence (i.e., psuedoreplication) of individuals within cages.

Among bees reared individually, we found no evidence of parasite virulence within the 8 d duration of the experiment. In *O. lignaria,* there was some evidence of lower probability of death among sham-inoculated bees in experiments with *C. mellificae* (χ^2_1 = 6.02, P = 0.014). However, this result is not significant at the Bonferroni-adjusted critical p-value of 0.0031. In *H. ligatus,* there was a non-significant trend of elevated mortality due to *N. ceranae* inoculation (χ^2_1 = 3.63, P = 0.057), with 4 of 16 (25%) bees dying in the parasite inoculation treatment, compared to 0 of 8 in the sham inoculation treatment.

SUPPLEMENTARY TABLES

Supplementary Table 1. Sample sizes by sex for *Halictus ligatus* and *Osmia lignaria* in the factorial cross-infection experiment. "N.male" indicates number of males; "N.female" indicates number of females. Only bees included in the analysis of infection are counted.

Host	Parasite	Treatment	N.male	N.female
Halictus	C. bombi	Parasite	5	12
Halictus	C. mellificae	Parasite	6	9
Halictus	L. passim	Parasite	3	14
Halictus	N. ceranae	Parasite	1	12
Osmia	C. bombi	Parasite	24	1
Osmia	C. mellificae	Parasite	6	5
Osmia	L. passim	Parasite	32	1
Osmia	N. ceranae	Parasite	5	13
Halictus	C. bombi	Sham	4	5
Halictus	C. mellificae	Sham	4	2
Halictus	L. passim	Sham	1	8
Halictus	N. ceranae	Sham	0	8
Osmia	C. bombi	Sham	17	0
Osmia	C. mellificae	Sham	2	0
Osmia	L. passim	Sham	22	1
Osmia	N. ceranae	Sham	4	4

Supplementary Table 2. Summary of infection prevalence and intensity in the factorial cross-infection experiment. "N.infected" indicates number of bees with detectable parasites. "N.total" refers to total sample size. "Median", "Q1", "Q3" and "Max" refer to the first, second, third and fourth quartiles of infection intensity, measured in parasite cell equivalents (*C. bombi, C. mellificae*, and *L. passim*) or gene copy equivalents (*N. ceranae)*.

Parasite	Host	Treatment	N.infected	N.total	Prevalence	Median	Q1	Q3	Max
C. bombi	Apis	Parasite	19	20	0.95	17.75536	3.759225	26.9945	95.97232
C. bombi	Apis	Sham	0	9	0	0	0	0	0
C. mellificae	Apis	Parasite	13	18	0.722222	77.60749	0.904866	8315.237	1097678
C. mellificae	Apis	Sham	6	11	0.545455	2.599262	0	8.385428	39.72839
L. passim	Apis	Parasite	8	21	0.380952	0	0	3.914429	372808.3
L. passim	Apis	Sham	2	10	0.2	0	0	0	3.006219
N. ceranae	Apis	Parasite	15	19	0.789474	3568.614	184.8659	1392441	64516646
N. ceranae	Apis	Sham	5	12	0.416667	0	0	1968.331	14148.89
C. bombi	Bombus	Parasite	11	17	0.647059	1578.251	0	22886.94	141811.8
C. bombi	Bombus	Sham	3	11	0.272727	0	0	1.183802	177.5985
C. mellificae	Bombus	Parasite	5	22	0.227273	0	0	0	3210.536
C. mellificae	Bombus	Sham	1	10	0.1	0	0	0	1608.868
L. passim	Bombus	Parasite	0	19	0	0	0	0	0
L. passim	Bombus	Sham	2	9	0.222222	0	0	0	0.791268
N. ceranae	Bombus	Parasite	2	18	0.111111	0	0	0	209.467
N. ceranae	Bombus	Sham	0	8	0	0	0	0	0
C. bombi	Halictus	Parasite	15	17	0.882353	695.6763	10.36213	16433.38	76692.13
C. bombi	Halictus	Sham	9	9	1	2.441564	0.416659	3.968759	10.04181
C. mellificae	Halictus	Parasite	15	15	1	2242120	1188908	2672216	7562375
C. mellificae	Halictus	Sham	6	6	1	46.52662	37.72481	98.36962	339.3173
L. passim	Halictus	Parasite	13	17	0.764706	7.198937	3.347586	38.29176	1048.755
L. passim	Halictus	Sham	1	9	0.111111	0	0	0	821.2235
N. ceranae	Halictus	Parasite	4	13	0.307692	0	0	19.10056	909.2322
N. ceranae	Halictus	Sham	0	8	0	0	0	0	0
C. bombi	Osmia	Parasite	13	25	0.52	0.478205	0	36686.14	1080462
C. bombi	Osmia	Sham	2	17	0.117647	0	0	0	15904.57
C. mellificae	Osmia	Parasite	10	11	0.909091	8316.307	1798.084	690186.3	3555330
C. mellificae	Osmia	Sham	0	2	0	0	0	0	0
L. passim	Osmia	Parasite	5	33	0.151515	0	0	0	551.3706
L. passim	Osmia	Sham	4	23	0.173913	0	0	0	8.289798
N. ceranae	Osmia	Parasite	9	18	0.5	24.70667	0	2093.752	14220.84
N. ceranae	Osmia	Sham	0	8	0	0	0	0	0

SUPPLEMENTARY METHODS

DNA cleanup and concentration

For *A. mellifera* and *B. impatiens* samples that failed to amplify in the Apidae PCR, guanidine contamination was suspected based on spectrophotometry and gel images from qualitative PCR. These samples were subjected to DNA cleanup with the DNEasy Powerclean Pro kit (Qiagen). Cleanup was conducted as instructed by the manufacturer: Half of the DNA extract (100 µL) was mixed with proprietary kit solutions "CU" and "IR" (50 µL each), vortexed, and centrifuged (2 min, 13000 g). The supernatant was transferred to a separate tube, mixed with kit solution "'SB" (400 µL), and centrifuged (1 min, 10000 g) through a DNA-binding spin column. The spin column was washed twice by addition of solution "CB" (500 µL) followed by centrifugation (30 s, 10000 g), followed by an additional centrifugation (2 min, 13000 g) to dry the column. The column-bound DNA was eluted by centrifugation (1 min, 10000 g) to the original volume (100 µL) in kit elution buffer (solution "EB") and stored at -80 °C.

For *O. lignaria* inoculated with *C. bombi* and *L. passim,* initial PCR assays likewise failed to amplify host DNA, and the concentration of DNA in the extract was low when measured by spectrophotometric and fluorescence-based methods (Qubit, Thermo Fisher, Waltham, MA). Accordingly, the extracted DNA was concentrated using ethanol precipitation. The DNA extract was mixed with a 5 M NaCl solution (4 µL per 100 µL total volume, final concentration 0.2 M NaCl), then precipitated by a 30 min incubation on ice with two volumes of 70% ethanol. The precipitate was centrifuged for 10 min at 12000 g and the supernatant removed. The resulting pellet washed with 70% ethanol to remove excess NaCl, then air-dried overnight before resuspension in Qiagen elution buffer "TE" to 10% of the initial volume (20 µL). Any samples for which Apidae PCR remained unsuccessful were excluded from the analysis of infection.

Molecular quantification of infection

Infection with each experiment's focal parasite was quantified by qPCR, with quantities corrected for DNA concentration (i.e., ethanol precipitation of *Osmia* samples) where appropriate. *Crithidia bombi* infection was quantified as previously described (Ulrich *et al.*, 2011; Palmer-Young *et al.*, 2018). Reactions were run in triplicate with primers for the *C. bombi* 18s rRNA gene ('CriRTF2' (GGCCACCCACGGGAATAT) and 'CriRTR2' (CAAAGCTTTCGCGTGAAGAAA)) (Ulrich *et al.*, 2011). The assay used 20 µL reaction volume (2 µL DNA extract and 300 nM of each primer in 1x SYBR Green Supermix (BioRad, Hercules, CA)). Thermocycler conditions included 10 min initial denaturation at 95 °C followed by 40 cycles of denaturation (15 s at 95 °C) and annealing-extension (60 s at 60 °C). Absolute quantifications (number of parasite cell equivalents) were made relative to a standard curve consisting of 7 dilutions of *C. bombi* DNA (equivalent of $3.9 * 10^3$ to $2.5 * 10^5$ cells) extracted from an aliquot of cell culture containing 10^6 parasite cells. The standards were run in triplicate on each assay plate, along with three no-template controls. Amplification efficiencies were checked to ensure they were between 90 and 110% on each plate; otherwise, the entire plate was repeated until the desired efficiency was achieved. *Crithidia mellificae* and *L. passim* infections were quantified using the same primers, thermocycler conditions, standards, and technical replication used for *C. bombi*. Cycle times were converted to parasite cell quantities based on standard curves, derived from the DNA extract of cell cultures of the appropriate species.

For *Nosema ceranae,* infection was quantified using primers specific to *Nosema ceranae* and excluding *N. apis* (NcF (AAGAGTGAGACCTATCAGCTAGTTG) and NcR (CCGTCTCTCAGGCTCCTTCTC)) (Bourgeois *et al.*, 2010; Rubanov *et al.*, 2019). Reactions were run in triplicate in 15 µL volume (3 µl template DNA and 200 nM of each primer in 1x SsoAdvanced Universal SybR Green Supermix). The thermocycler conditions were: 95 °C for 3 minutes followed by 40 cycles of 95 °C for 10 seconds and

58 °C for 30 seconds. Cycle times were converted to copy numbers based on a standard curve made by amplification of a purified plasmid (Rubanov *et al.*, 2019). The standards were made by cloning NcF/NcR PCR product from the gut extract of an infected bee into *E. coli* using the TopoTA cloning kit (Rubanov *et al.*, 2019). Plasmids were purified with a plasmid purification kit (Purelink, Invitrogen, Carlsbad, CA) and linearized with the Pst1 restriction enzyme (New England Biolabs, Ipswich, MA). Concentration of plasmids in the stock solution of linearized plasmid were estimated by fluorescence-based quantification of DNA concentration using a Qubit (Invitrogen). Seven standards (10-fold dilutions from 10^8 to 10^2 copies per reaction) and a no template control were run in triplicate on each plate. Amplification efficiencies were checked to ensure they were between 90 and 110% on each plate.

KEY TO SUPPLEMENTARY DATA

***Crithidia bombi-Megachile* experiment data**

"Cbombi_Megachile_data.csv"

Variables:

Bee: Unique numeric ID for each individual

Host: Host (bee) species

Sex: Host sex

Trial: Experiment run. The first trial was conducted with *Megachile rotundata* males, the second with *Megachile rotundata* females.

Date.inoc: Date inoculated

Method.inoc: Method used to prepare inoculum

Date.death: Date observed dead

Date.dissect: Date dissected

Count.crith: Parasite cell count in 0.02 microliters gut homogenate

Tot.crith: Total number of parasite cells per host, extrapolated from parasite cell count

Fold_increase: Fold change in infection intensity relative to quantity inoculated (6000 cells for *Megachile rotundata,* 12000 cells for *Bombus impatiens)*

Distance.intertegular: Intertegular distance (in mm, for *Megachile rotundata* only)

Factorial cross-infection experiment: Infection

“Infection_data.csv”

Variables:

Year: Year of experiment

Parasite: Parasite tested in corresponding week of experiment

Host: Host bee species

Colony: Colony of origin (for *Bombus impatiens* only)

Treatment: Whether bee was inoculated with Parasite or parasite-free Sham control

Number: Unique number of individual bee within each host-parasite treatment combination

Parasite.quantity: Infection intensity, measured in parasite cell equivalents for the three trypanosomatids *Crithidia bombi*, *Crithidia mellificae*, and *Lotmaria passim*; or in parasite gene copy equivalents for *Nosema ceranae*

Sex: Host sex

DNA_concentrated: Whether DNA was subject to ethanol precipitation due to low initial concentration

Concentration_multiplier: Correction factor applied in calculation of 'Parasite.quantity', to account for reduction in sample volume during DNA precipitation

Factorial cross-infection experiment: Mortality

“Mortality_data.csv”

Variables:

Year: Year of trial. Infections of *Osmia lignaria* with *C. bombi* and *C. mellificae* were conducted in 2019 due to low availability of bees in 2018.

Host: Host bee species

Parasite: Parasite used in the corresponding block of the experiment

Colony: Colony of origin (for *Bombus impatiens* only)

Treatment: Whether bee was inoculated with Parasite or parasite-free Sham control

Number: Unique number of individual bee within each host-parasite treatment combination

Dead_before: Whether bee died before end of trial (8 d post-inoculation)

End_Time: Number of days from inoculation until removal from experiment due to death, escape, or dissection

REFERENCES

Bourgeois, A. L., Rinderer, T. E., Beaman, L. D. and Danka, R. G. (2010). Genetic detection and quantification of Nosema apis and N. ceranae in the honey bee. *Journal of Invertebrate Pathology* **103**, 53–58. doi: 10.1016/j.jip.2009.10.009.

Palmer-Young, E. C., Calhoun, A. C., Mirzayeva, A. and Sadd, B. M. (2018). Effects of the floral phytochemical eugenol on parasite evolution and bumble bee infection and preference. *Scientific Reports* **8**, 2074. doi: 10.1038/s41598-018-20369-2.

Rubanov, A., Russell, K. A., Rothman, J. A., Nieh, J. C. and McFrederick, Q. S. (2019). Intensity of Nosema ceranae infection is associated with specific honey bee gut bacteria and weakly associated with gut microbiome structure. *Scientific Reports* **9**, 3820. doi: 10.1038/s41598-019-40347-6.

Ulrich, Y., Sadd, B. M. and Schmid-Hempel, P. (2011). Strain filtering and transmission of a mixed infection in a social insect. *Journal of evolutionary biology* **24**, 354–62. doi: 10.1111/j.1420-9101.2010.02172.x.
